# Minocycline and antipsychotics inhibit inflammatory responses in BV-2 microglia activated by LPS via regulating the MAPKs/ JAK-STAT signaling pathway

**DOI:** 10.1186/s12888-023-05014-1

**Published:** 2023-07-18

**Authors:** Yujun Long, Ying Wang, Yidong Shen, Jing Huang, Yamin Li, Renrong Wu, Jingping Zhao

**Affiliations:** 1 Department of Psychiatry, National Clinical Research Center for Mental Disorders, and National Center for Mental Disorders, The Second Xiangya Hospital of Central South University, Changsha, Hunan 410011 China; 2 Mental Health Center of Xiangya Hospital, Central South University, Changsha, Hunan China

**Keywords:** Antipsychotics, LPS, Minocycline, Risperidone, Haloperidol, MAPKs, JAK-STAT

## Abstract

**Background:**

Abnormal activation of microglia is involved in the pathogenesis of schizophrenia. Minocycline and antipsychotics have been reported to be effective in inhibiting the activation of microglia and thus alleviating the negative symptoms of patients with schizophrenia. However, the specific molecular mechanism by which minocycline and antipsychotics inhibit microglial activation is not clear. In this study, we aimed to explore the molecular mechanism of treatment effect of minocycline and antipsychotics on schizophrenia.

**Methods:**

Microglia cells were activated by lipopolysaccharide (LPS) and further treated with minocycline, haloperidol, and risperidone. Then cell morphology, specific marker, cytokines, and nitric oxide production process, and the proteins in related molecular signaling pathways in LPS-activated microglia were compared among groups.

**Results:**

The study found that minocycline, risperidone, and haloperidol significantly inhibited morphological changes and reduced the expression of OX-42 protein induced by LPS. Minocycline significantly decreased the production of interleukin-6 (IL-6), tumor necrosis factor-alpha (TNF-α), and interleukin-1beta (IL-1β). Risperidone also showed significant decrease in the production of IL-6 and TNF-α, while haloperidol only showed significant decrease in the production of IL-6. Minocycline, risperidone, and haloperidol were found to significantly inhibit nitric oxide (NO) expression, but had no effect on inducible nitric oxide synthase (iNOS) expression. Both minocycline and risperidone were effective in decreasing the activity of c‑Jun N‑terminal kinase (JNK) and extracellular signal-regulated kinase (ERK) in the mitogen-activated protein kinases (MAPKs) signal pathway. Additionally, minocycline and risperidone were found to increase the activity of phosphorylated-p38. In contrast, haloperidol only suppressed the activity of ERK. Minocycline also suppressed the activation of janus kinase 2 (JAK2) and signal transducer and activator of transcription 3 (STAT3), while risperidone and haloperidol only suppressed the activation of STAT3.

**Conclusions:**

The results demonstrated that minocycline and risperidone exert stronger anti-inflammatory and neuroprotective effects stronger than haloperidol, through MAPKs and Janus kinase-signal transducer and activator of transcription (JAK-STAT) signaling pathways in BV2 cells stimulated with LPS, revealing the underlying mechanisms of minocycline and atypical antipsychotics in the treatment of negative schizophrenia symptoms.

**Supplementary Information:**

The online version contains supplementary material available at 10.1186/s12888-023-05014-1.

## Background

Schizophrenia is a neurodevelopmental disease characterized by positive symptoms, negative symptoms and cognitive symptoms. The mechanisms of schizophrenia have not yet been elucidated. Microglia are neuroprotective, immunocompetent cells of the central nervous system (CNS) that play a pivotal role in neurodevelopmental processes [1, 2]. Accumulating evidence has shown that abnormal activation of microglia is involved in the pathogenesis of schizophrenia by facilitating the release of proinflammatory cytokines and various free radicals, leading to developmental abnormalities [3–5]. Clinical studies have found that anti-inflammatory drugs that can inhibit the abnormal activation of microglia can alleviate the negative symptoms of schizophrenia. In addition, previous studies have found that atypical antipsychotics, which have been found to be effective in treating negative symptoms, also inhibit the activation of microglia, suggesting that atypical antipsychotics also ameliorate the negative symptoms of schizophrenia by inhibiting the abnormal activation of microglia.

Microglial activation is manifested by changes in cell morphology (retraction of processes and hypertrophy), changes in the expression of specific cell surface markers (OX-42, a complement type III receptor) and the release of some harmful substances (proinflammatory cytokines, free radicals, etc.). There are a wide range of factors that trigger the activation of microglia, such as LPS, interferon alpha (INF-α), and β-amyloid (Aβ). When microglia are stimulated, they are exposed to a series of signals, leading to the expression of related inflammatory factors and thereby regulating the inflammatory response [1]. Studies have found that some signaling molecules involved in the activation process of microglia are related to mental illness. Studies have found that the Janus kinase-signal transducer and activator of transcription (JAK-STAT) signaling pathway is involved in synaptic plasticity in the hippocampus and related to depression [6–9]. A study involving a model of neuroinflammation found that the atypical antipsychotic risperidone inhibited the increase in p38 mitogen-activated protein kinases (MAPK) expression induced by LPS [10]. Based on the above results, we believe that these two signaling pathways are likely related to the therapeutic effects of antipsychotics.

Minocycline is a tetracyclic antibiotic that exerts anti-inflammatory and neuroprotective effects by inhibiting the activation of microglia [11]. Previous study [12] have shown that the MAPK, and JAK-STAT signaling pathways are involved in the anti-inflammatory effect of minocycline. Clinical evidence has shown that when used as an adjunct therapy, minocycline can alleviate the negative symptoms of schizophrenia better than placebo [13]. Some in vitro and in vivo studies have found that minocycline and atypical antipsychotics can inhibit the activation of microglia, while typical antipsychotics have little effect on microglial activation [14, 15]. Our previous study also found that minocycline and risperidone can significantly rescue behavioral deficits and attenuate microglial activation in rat models of schizophrenia [16, 17]. Haloperidol is a typical antipsychotic used for the treatment of positive symptoms, and its inhibitory effect on microglial activation is significantly weaker than that of risperidone [18].

Although the “Microglia hypothesis of schizophrenia” has attracted much attention, the molecular mechanism by which atypical antipsychotics inhibit microglia activation is still unclear [19, 20]. Therefore, the aim of this study was to explore the molecular mechanism of treatment effect of minocycline and antipsychotics on schizophrenia through observation of related molecular signaling pathways in activated microglia. Our research hypothesis is that atypical antipsychotic risperidone, similar to minocycline, inhibits the activation of microglia through the MAPK, and JAK-STAT signaling pathways, thereby exerting a therapeutic effect on the negative symptoms of schizophrenia. The inhibitory effect of typical antipsychotic haloperidol on MAPK, and JAK-STAT signaling pathway and microglia activation is significantly weaker than that of minocycline and risperidone. This study provides evidence for the pathogenesis of negative schizophrenia symptoms and new targets for treatment.

## Methods

### Drugs and reagents

Minocycline hydrochloride, risperidone, haloperidol and LPS were purchased from Sigma Chemical Co. (St. Louis, MO, USA, catalog no. WXBB4793V, R3030, H1512, and 025M4040V, respectively). RPMI 1640 medium, fetal bovine serum, penicillin/streptomycin antibiotics and 0.25% trypsin-EDTA were purchased from Gibco BRL (Grand Island, NY, USA, catalog no. 11875119, 10091148, 15140148, and 25200072, respectively). The measurement of nitric oxide using the Nitrite/Nitrate Assay Kit purchased from Sigma-Aldrich (St. Louis, MO, USA, catalog no. 23479-1KT-F). Rabbit anti-OX42 Polyclonal Antibody was purchased from Absin Bioscience Inc. (Shanghai, China, catalog no. abs137035). Sheep Anti-Rabbit IgG H&L (DyLight® 488) was purchased from Abcam Bioscience Inc. (Cambridge, UK, catalog no. ab96923). Mouse TNF-α, IL-1β ELISA kits were from R&D Systems, Inc. (NE Minneapolis, USA, catalog no. MTA00B, and MLB00C, respectively). Mouse IL-6 ELISA kits purchased from CUSABIO (Fannin St., Houston, USA, catalog no.CSB-E04639m). P38 MAPK (D13E1) XP ®Rabbit mAb, Phospho-p38 MAPK (Thr180/Tyr182) (D3F9) XP ®Rabbit mAb, iNOS (D6B6S) Rabbit mAb, JNK Rabbit mAb, ERK Rabbit mAb antibody, and α-Tubulin Rabbit mAb antibodies were purchased from CST (Boston, USA, catalog no. 8690, 4511, 13120, 67096, 4695, and 3873, respectively), STAT3 Rabbit mAb and JAK2 Rabbit mAb antibodies were purchased from Novus (Colorado, USA, catalog no. NBP2-61588, and NBP3-15825).

### BV-2 cell culture

The BV2 murine microglial cells were gifted from Shanghai Institutes for Biological Sciences (SIBS, Shanghai, China). The cells were cultured in RPMI 1640 medium supplemented with 10% fetal bovine serum in a 5% CO_2_ cell incubator at 37 °C. The cells were subcultured when the cell monolayer reach approximately 80% confluence.

### Viability assay

BV-2 cell viability was measured by the CCK-8 assay. In brief, BV-2 cells (100 μL) were seeded in each well of a 96-well culture plate (0.5 × 10^4^ cells/mL). Twenty-four hours later, different concentrations of drug-containing and serum-free medium were added. The cells were treated with different concentrations of minocycline (0.01, 0.1, 1.0, 10, and 100 μmol/L), risperidone (0.1, 1.0, 10, 50 and 100 μmol/L) and haloperidol (0.1, 1.0, 10, 50 and 100 μmol/L); normally cultured cells were established as controls for each experimental group. Cells from each group were plated in 3 wells of a 96-well culture plate and cultured at 37 °C and 5% CO_2_ for 24 h. Then, 10 μL of CCK-8 reagent was added to each well for 3 h, and the absorbance (OD) at 450 nm was measured using a microplate reader connected to an enzyme labeling instrument. The relative survival rate of the cells was calculated as follows:

$$\begin{array}{*{20}{c}}{{\rm{\% }}\,{\rm{cell}}\,{\rm{viability}}\,{\rm{ = }}\,{\rm{(O}}{{\rm{D}}_{{\rm{experimental}}\,{\rm{group}}}}{\mkern 1mu} {\rm{ - }}{\mkern 1mu} {\rm{O}}{{\rm{D}}_{{\rm{blank}}\,{\rm{group}}}}{\rm{)}}}\\{{\rm{/(O}}{{\rm{D}}_{{\rm{normal}}\,{\rm{group}}}}{\mkern 1mu} {\rm{ - }}{\mkern 1mu} {\rm{O}}{{\rm{D}}_{{\rm{blank}}\,{\rm{group}}}}{\rm{)}}{\mkern 1mu} {\rm{ \times }}{\mkern 1mu} {\rm{100\% }}}\end{array}$$

After selecting the most effective concentrations of each drug, the optimal LPS treatment duration was selected. The experiment was divided into three parts to investigate the effects of minocycline, risperidone and haloperidol on cell viability. In each part of the experiment, the cells were divided into three groups: the normal culture group, LPS treatment group and drug pretreatment plus LPS treatment group. Cells from each group were plated in three wells in each part of the experiment, and the 96-well culture plate was cultured at 37 °C for 24 h in 5% CO_2_. The cells were treated with the optimal concentrations of the three drugs for 24 h and then treated with 1.0 μg/ml LPS for 12, 24 or 48 h. After incubation for 3 h with 10 μL of CCK-8 reagent, the OD value of each well was measured by an enzyme labeling instrument, and the optimal LPS treatment duration was determined by calculating the relative survival rate of the cells in each well.

### Morphological observation

The BV2 cells were plated into 6-well plates at a density of 5 × 10^4^cells per well. After the cells had adhered to the wall overnight, they were randomly divided into 5 groups: (1) the blank control group; (2) the LPS group; (3) the minocycline preconditioning + LPS group; (4) the risperidone preconditioning + LPS group; and (5) the haloperidol preconditioning + LPS group. The cells in each group were photographed under a phase contrast microscope at the end of the treatment period to observe the effects of the drugs on the activation of cells stimulated with LPS. The number of branches and the branch length of each cell were measured using ImageJ.

### Immunofluorescence staining and measurement of mean fluorescence intensity

The protein expression of OX42 (CD11b/c), a microglia-specific marker, in BV-2 cells was assessed by immunofluorescence, and changes in its expression were observed. After the cells in the five groups were digested with 0.25% trypsin, they were inoculated onto slides at a density of 1 × 10^5^ cells/ml and washed with PBS. After that, the cells were fixed with 4% paraformaldehyde in PBS, permeabilized with 0.1% Tween in PBS (PBST) and blocked with a 5% BSA working solution. After being diluted 1:100, the first antibody was added to the cells and incubated at 4 °C overnight. The cells in the negative control group were treated with PBS rather than the primary antibody. The next day, the cells were allowed to recover at 37 °C for 45 min, washed with PBS, and then incubated with a sheep anti-rabbit secondary antibody (diluted 1:1000) at room temperature for 30 min. Finally, after washing with PBS, the slides were sealed with glycerin and photographed under a microscope. For relative fluorescence analysis, all settings such as condenser opening, objective, zoom, exposure time, and gain parameters were maintained constant for all samples.

Initially fluorescence images were converted to Lab Stack and then split into single channel images. 32-bit images of single channel were converted into 8-bit images. The gray value of each pixel represents the fluorescence intensity of the point. Mean fluorescence intensity (MFI), mean gray value, was calculated using integrated intensity per unit area. A threshold was then chosen using the ‘Auto threshold’ function of ImageJ. Furthermore, the image was processed with the Analyze Particles algorithm of ImageJ to determine the number of single fluorescence cells computationally. The mean fluorescence intensity of single-cell was quantified in ImageJ using the ROI (region of interest) toolbox.

### Measurement of nitric oxide and inflammatory factors production

The BV-2 microglial cells in the logarithmic growth phase were suspended and seeded in 6-well culture plates (10 × 10^4^ cells/mL). The cells were randomly divided into 5 groups. After 24 h, the drugs at the appropriate concentrations, as determined by the CCK-8 assay, were added to the three drug groups, while serum-free medium was added to the blank control and LPS groups. The cells in the five groups were incubated for 24 h. All groups except the blank control group were stimulated with 1 μg/mL LPS for 24 h. The cell supernatant was collected, and the level of NO in the medium was measured with a nitric oxide one-step kit. The levels of IL-1β, IL-6 and TNF-α in the cell supernatant were measured by ELISA kits according to the manufacturer’s instructions.

### Western blot analysis

After discarding the culture media of the five groups of BV-2 cells, the cells were washed with PBS twice, and 2 μL of the protease inhibitor PMSF and 200 μL of protein lysate were added. After lysis for 30 min, the cells were centrifuged; the precipitate was discarded, and the supernatant was collected. The proteins were separated by SDS-PAGE (10%) and then transferred onto PVDF membranes. The membranes were incubated with specific antibodies (anti-iNOS, anti-p38, anti-pho-p38, anti-JNK, anti-ERK, anti-JAK-2, anti-STAT3 and anti-α-Tubulin) diluted 1:2000 overnight at 4 °C. The membrane was then washed with TBST, destained, dried, and incubated with a secondary antibody diluted 1:5000 at 37 °C for 2 h. Using α-Tubulin as an internal reference, the relative densities of the bands were analyzed by ImageJ software.

### Statistical analysis

Each experiment was repeated more than three times, and the experimental results were expressed as mean ± standard error. The comparison between two groups was compared by one-way analysis of variance (ANOVA) followed by the LSD post-hoc test using SPSS 23.0 software, *p* < 0.05 was considered to be statistically significant.

## Results

### BV-2 cell viability

**Fig. 1:**
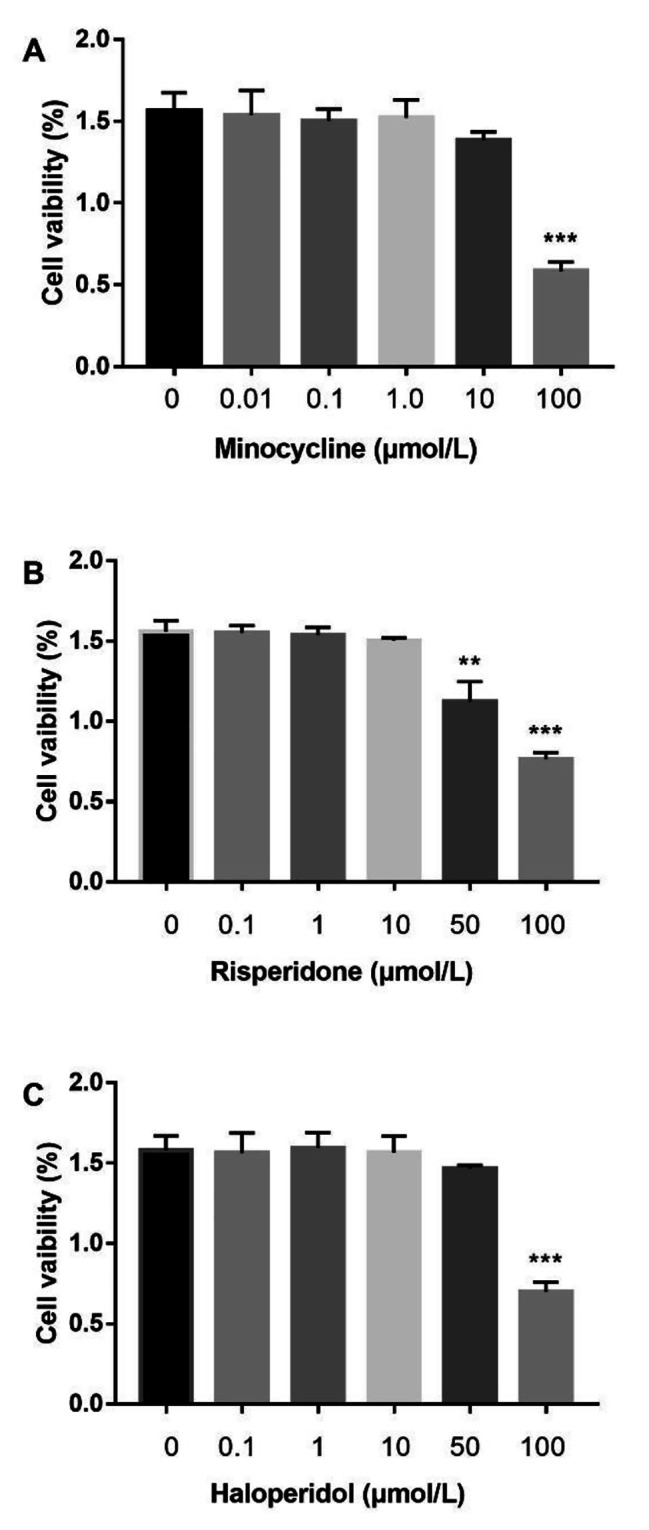
Effects of minocycline, risperidone and haloperidol on viability of BV-2 cells. Cells were treated with minocycline (**A**) at concentrations of 0.01–100 μmol/L or risperidone (**B**) at concentrations of 0.1–100 μmol/L or haloperidol (**C**) at concentrations of 0.1–100 μmol/L for 24 h. The cell viability was measured by CCK-8 assay. Each column represents the results of three independent experiments. ** *p* < 0.01 and *** *p* < 0.001 was used as statistical significance to compare with the untreated control.

To determine the appropriate concentrations of minocycline, risperidone and haloperidol, their cytotoxicity to BV-2 microglia was assessed by treating the cells with different concentrations of the drugs for 24 h and then measuring cell viability by the CCK-8 assay. The results showed that minocycline at a concentration of 10 μmol/L, risperidone at concentrations of 50 and 100 μmol/L and haloperidol at a concentration of 100 μmol/L significantly decreased the viability of BV-2 cells, while there was no significant change in cell viability after treatment with the other concentrations of the drugs (Fig. 1). Therefore, we chose 10 μmol/L, 10 μmol/L and 50 μmol/L as the appropriate concentrations of minocycline, risperidone and haloperidol, respectively, for subsequent experiments.

**Fig. 2:**
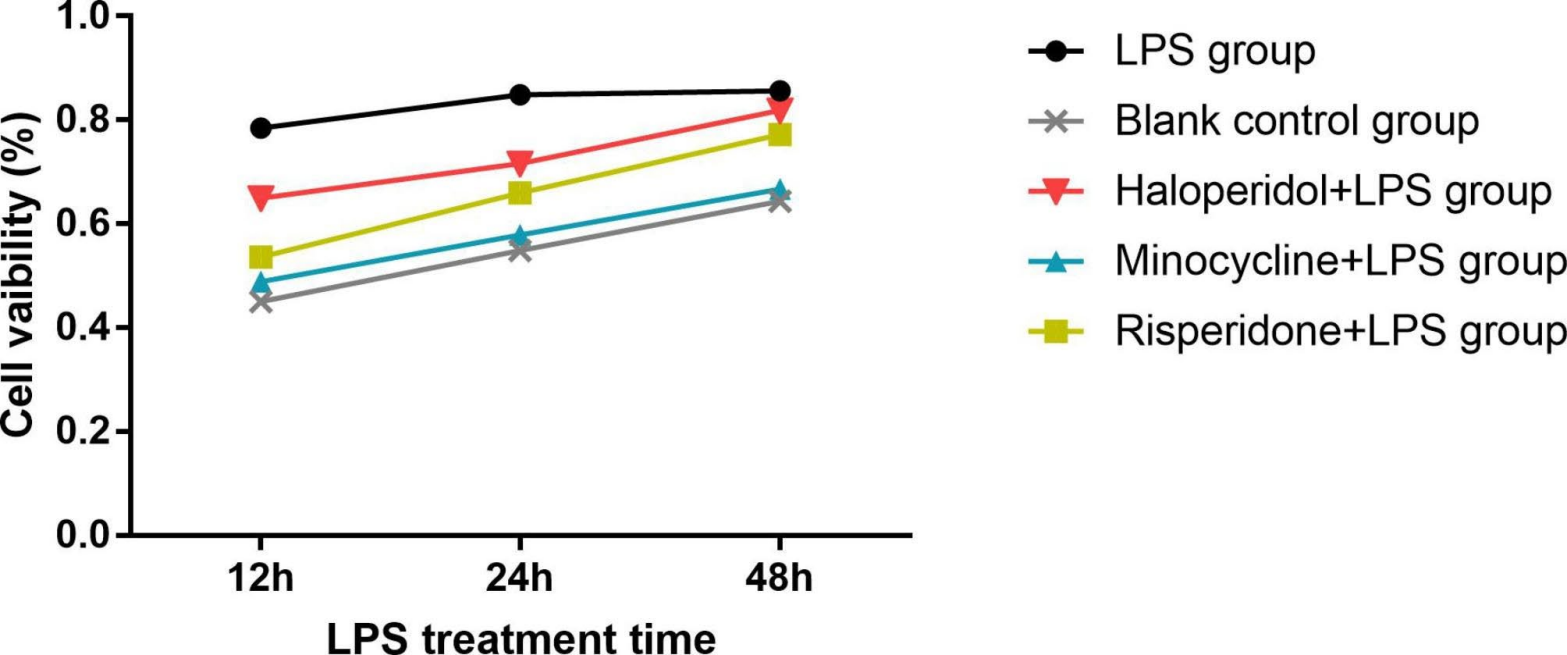
Effects of different LPS treatment time on viability of BV-2 cells. Cells were divided into five groups. The blank control group was treated with nothing. The three drug groups were pretreated with 10 μmol/L minocycline, 10 μmol/L risperidone and 50 μmol/L haloperidol respectively for 24 h. Then, the LPS group and three drug groups were treated with 1.0 ug/ml LPS for 12, 24 and 48 h, respectively. The cell viability was measured by CCK-8 assay.

To determine the optimal duration of LPS treatment, BV-2 microglial cells were pretreated with the optimal concentrations of minocycline, risperidone and haloperidol for 24 h and then treated with LPS for 12 h, 24 or 48 h. The CCK-8 assay showed no significant differences in the viabilities of cells treated with the three drugs after LPS treatment for different amounts of time. However, in the LPS group, cell activity was decreased after 48 h of LPS treatment; thus, 24 h was chosen as the appropriate LPS treatment duration (Fig. 2).

### Effects of minocycline and antipsychotics on morphological changes of microglia activated by LPS

**Fig. 3:**
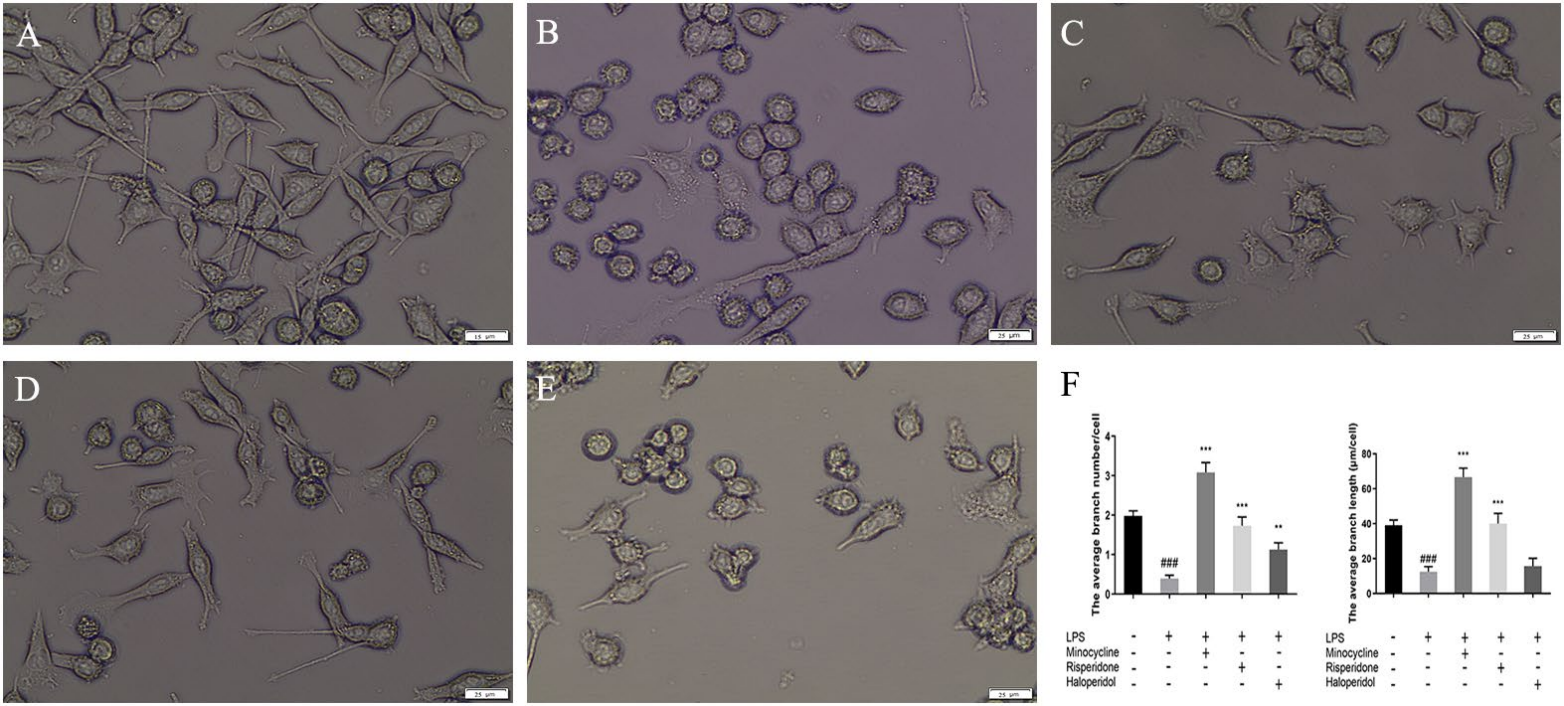
Effects of minocycline, risperidone and haloperidol on LPS induced morphological changes in BV2 cells. The cells were randomly divided into 5 groups. The blank control group (**A**) was treated with nothing, the LPS group (**B**) was treated with 1.0 ug/ml LPS for 24 h. The three drug groups were pretreated with 10 μmol/L minocycline (**C**), 10 μmol/L risperidone (**D**) and 50 μmol/L haloperidol (**E**) respectively for 24 h, and then 1.0 ug/ml LPS was added for 24 h. The morphology of BV2 cells was observed under 400X-ray light microscope. Each group chose one of the three separate images as a representative. Scale bar in Fig.**A** is 15 μm, in Fig.**B**-**E** is 25 μm. The bar chart (**F**) shows statistical results of WB. ^###^*p* < 0.001 was compared with the blank control group; **p < 0.01 and ***p < 0.001 were compared with the LPS group.

We directly observed the morphological characteristics of cells treated with different antipsychotics and LPS. As depicted in Fig. 3A, normal BV-2 cells possess slender processes and long branches, with an average branch number of 1.98 ± 0.13/cell and an average branch length of 39.14 ± 2.93 μm/cell. Following 24 h of LPS stimulation (Fig. 3B), the BV2 cells exhibited a round cell body and retracting processes, resulting in an amoeboid morphology with an average branch number of 0.39 ± 0.09/cell (*p* < 0.001, compared with the blank group) and an average branch length of 12.51 ± 2.91 μm/cell (*p* < 0.001, compared with the blank group). Pretreatment with minocycline (Fig. 3C) resulted in a noticeable increase in the number of branches per cell, with an average of 3.08 ± 0.25/cell (*p* < 0.001, compared with the LPS group), and an increase in the average branch length to 66.67 ± 5.09 μm/cell (*p* < 0.001, compared with the LPS group). Pretreatment with risperidone (Fig. 3D) increased the number of branches per cell to 1.73 ± 0.22 (*p* < 0.001, compared with the LPS group), with an increase in the average branch length to 40.03 ± 5.88 μm/cell (*p* < 0.001, compared with the LPS group). For BV-2 cells pretreated with haloperidol (Fig. 3E), the number of branches per cell increased to 1.13 ± 0.17 (*p* < 0.01, compared with the LPS group), whereas the average branch length did not change from the LPS group at 15.70 ± 4.47 μm/cell. The statistical results of the number of branches and average branch length were showed in Fig. 3F. These findings suggest that minocycline, risperidone, and haloperidol attenuate LPS-induced morphological changes in BV2 cells. Moreover, from our results, minocycline has the most significant effect in attenuating cell morphology (*p* < 0.001, compared with the risperidone and haloperidol groups), with risperidone exhibiting the second-most substantial effect (*p* < 0.05, compared with the haloperidol group, in terms of branch numbers and *p* < 0.001, compared with the haloperidol group, in terms of branch length), and haloperidol showing the weakest effect.

### Effects of minocycline and antipsychotics on OX42 marker of microglia activated by LPS

**Fig. 4:**
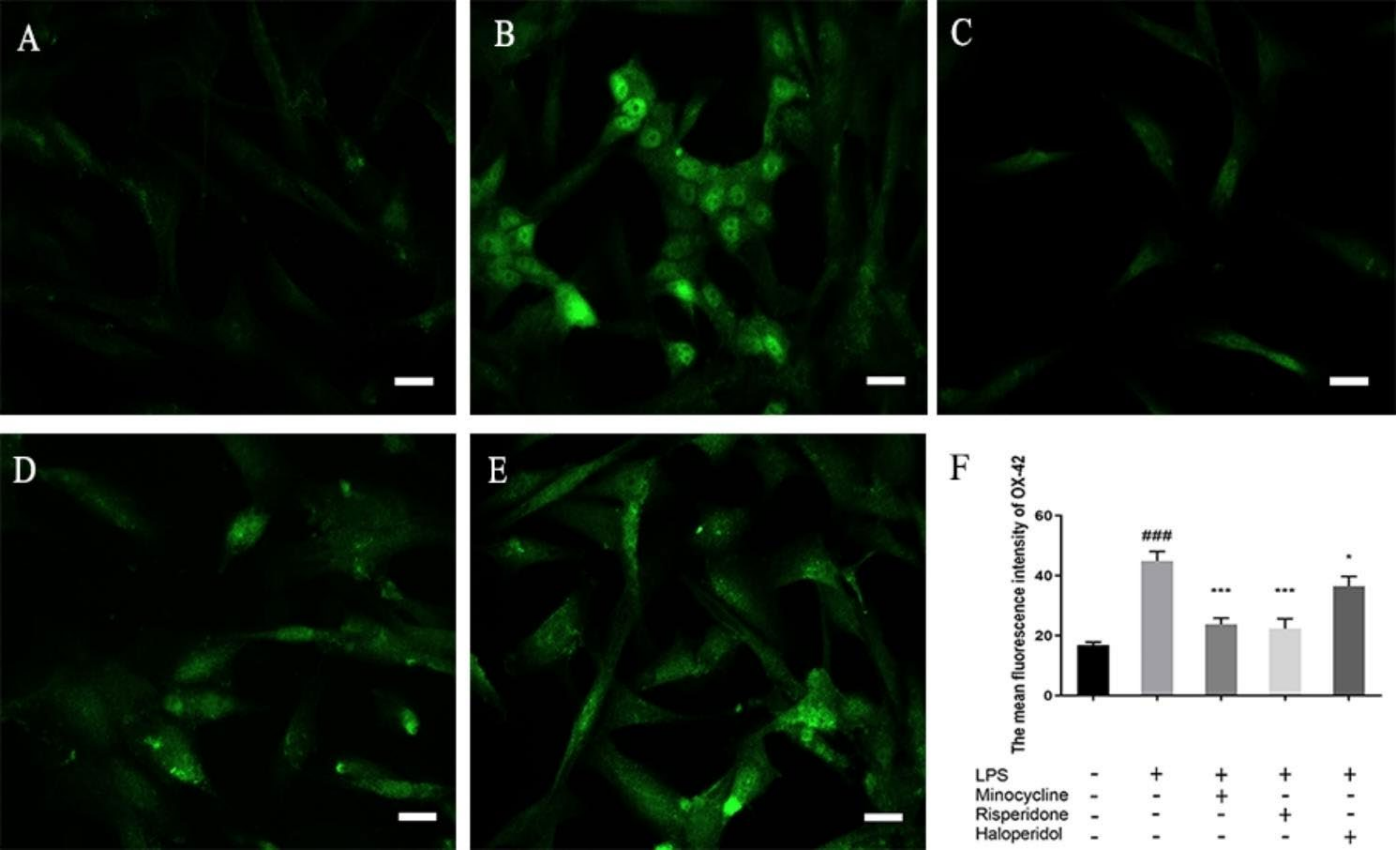
Effects of minocycline and antipsychotics on OX42 marker of microglia activated by LPS. The cells were randomly divided into 5 groups. The blank control group (**A**) was treated with nothing, the LPS group (**B**) was treated with 1.0 ug/ml LPS for 24 h. The three drug groups were pretreated with 10 μmol/L minocycline (**C**), 10 μmol/L risperidone (**D**) and 50 μmol/L haloperidol (**E**) respectively for 24 h, and then 1.0 ug/ml LPS was added for 24 h. The changes of microglia specific marker OX-42 were observed by immunofluorescence. Each group chose one of the three separate images as a representative. Scale bar in Fig.A is 15 μm, in Fig.B-E is 25 μm. The bar chart (**F**) shows statistical results of MFI. ^###^*p* < 0.001 was compared with the blank control group; *p < 0.05, **p < 0.01, and ***p < 0.001 were compared with the LPS group.

We measured the mean fluorescence intensity (MFI) of OX-42 treated with different antipsychotics and LPS. As showed in Fig. 4, the MFI of OX-42 in normal BV-2 cells was 16.85 ± 0.99. Following 24 h of LPS stimulation, the MFI of OX-42 was significantly higher at 44.91 ± 3.12 (*p* < 0.001, compared with the blank group). Pretreatment with minocycline significantly decrease the MFI of OX-42 to 23.78 ± 2.02 (*p* < 0.001, compared with the LPS group). Pretreatment with risperidone significantly decrease the MFI of OX-42 to 22.30 ± 3.26 (*p* < 0.001, compared with the LPS group). Pretreatment with haloperidol significantly decrease the MFI of OX-42 to 36.47 ± 3.27 (*p* < 0.05, compared with the LPS group). After pre-treatment with minocycline and risperidone, the MFI of OX-42 in LPS-activated BV-2 cells did not show any significant difference when compared to the blank group. However, pre-treatment with haloperidol resulted in a significantly higher MFI of OX-42 compared to the blank group (p < 0.001). The MFI of OX-42 in the risperidone group was not significantly different from the minocycline group (p = 0.724), and it was significantly lower than the haloperidol group (p < 0.001). Furthermore, the haloperidol group showed a significantly higher MFI than the minocycline group (p < 0.01). Overall, our data suggest that these drugs may have potential therapeutic effects in reducing microglial activation in neuroinflammatory conditions.

### Effects of minocycline and antipsychotics on inflammatory cytokines production of microglia activated by LPS

**Fig. 5:**
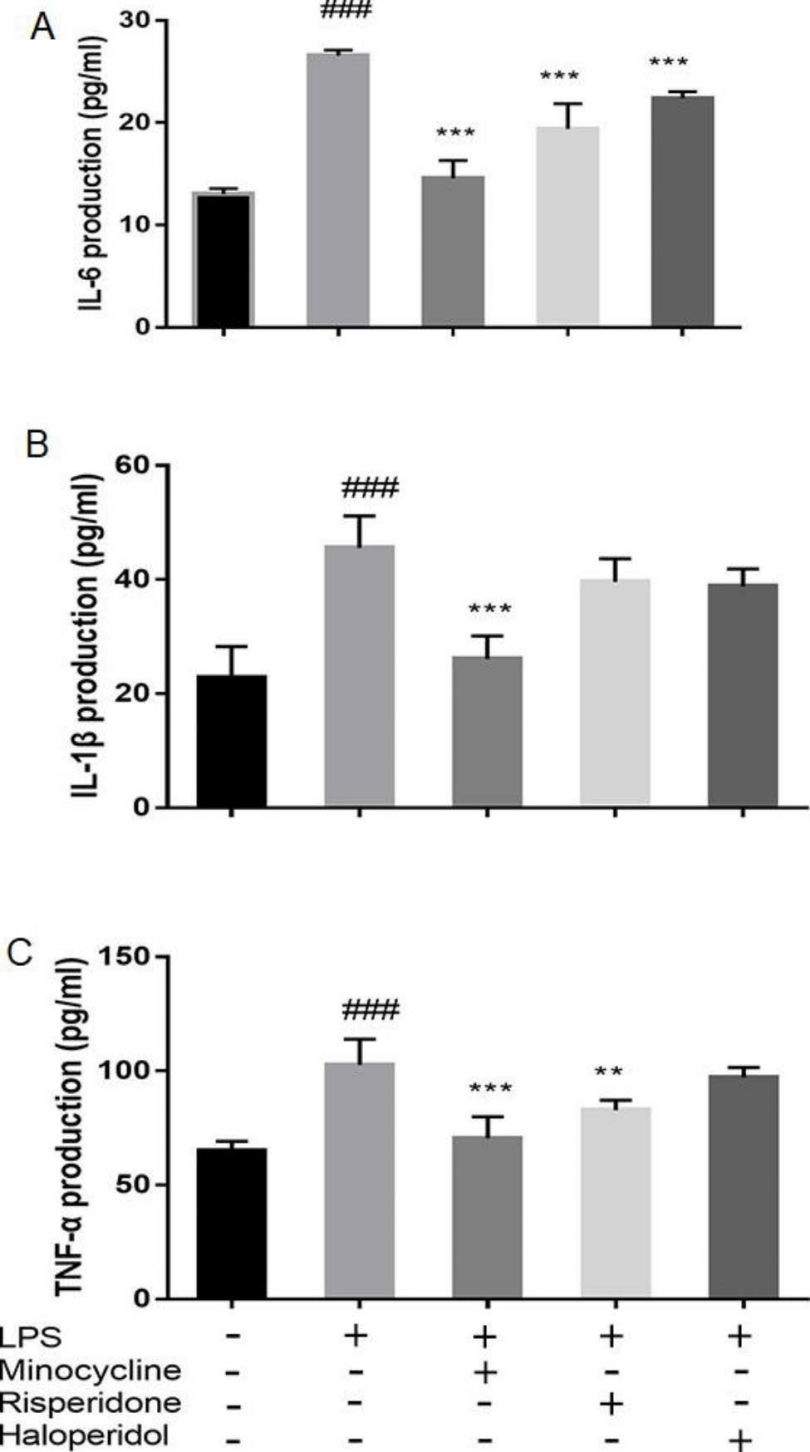
Effects of minocycline and antipsychotics on inflammatory cytokines production of microglia activated by LPS. The cells were randomly divided into 5 groups. The blank control group was treated with nothing, the LPS group was treated with 1.0 ug/ml LPS for 24 h. The three drug groups were pretreated with 10 μmol/L minocycline, 10 μmol/L risperidone and 50 μmol/L haloperidol respectively for 24 h, and then 1.0 ug/ml LPS was added for 24 h. Then, the conditioned medium was collected and the production of IL-6 (**A**), IL-1β (**B**), and TNF-α (**C**) was quantified by ELISA kit. The results were from six independent experiments. ^###^*p* < 0.001 was compared with the blank control group; **p < 0.01 and ***p < 0.001 were compared with the LPS group.

The concentration of interleukin-6 (IL-6) in normal BV-2 cells was found to be 13.04 ± 0.51 pg/ml, whereas it was significantly higher in BV-2 cells treated with LPS, with a concentration of 26.57 ± 0.51 pg/ml (*p* < 0.001). On pretreatment with minocycline and antipsychotics, the concentration of IL-6 significantly decreased. The concentration was found to be 14.56 ± 1.75 pg/ml with minocycline (*p* < 0.001), 19.39 ± 2.46 pg/ml with risperidone (*p* < 0.001), and 22.40 ± 0.65 pg/ml with haloperidol (*p* < 0.001). (Fig. 5A)

Similarly, the interleukin-1β (IL-1β) in normal BV-2 cells had a concentration of 22.83 ± 5.43 pg/ml, while LPS-treated BV-2 cells had a significantly higher concentration of 45.56 ± 5.62 pg/ml (*p* < 0.001). Pretreatment with minocycline resulted in a significant decrease in IL-1β concentration to 26.06 ± 4.05 pg/ml (*p* < 0.001). However, pretreatment with risperidone and haloperidol resulted in concentrations of 39.61 ± 4.06 pg/ml and 38.83 ± 3.03 pg/ml, respectively, which were not significantly different from the concentration in LPS-treated BV-2 cells. (Fig. 5B)

In the blank group, the levels of TNF-α were 65.00 ± 4.17 pg/ml. Compared to the blank group, the LPS group had significantly higher levels of TNF-α, measuring at 102.50 ± 11.39 pg/ml (*p* < 0.001). The minocycline group had TNF-α levels of 70.50 ± 9.41 pg/ml (*p* < 0.001), while the risperidone group had levels of 82.83 ± 4.34 pg/ml (*p* < 0.01), both significantly lower than the LPS group. The haloperidol group had TNF-α levels of 97.17 ± 4.45 pg/ml, which did not differ significantly from the concentration in LPS-treated BV-2 cells. (Fig. 5C)

### Effects of minocycline and antipsychotics on NO production and iNOS expression of microglia activated by LPS

Supernatants were collected from five groups and used in the experiment. Following treatment with LPS, the production of NO in BV2 cells significantly increased from 9.92 ± 2.04 μM/L to 47.33 ± 3.49 μM/L (*p* < 0.001). Pretreatment with minocycline resulted in a decrease in NO production to 16.25 ± 1.54 μM/L compared to the LPS group (*p* < 0.001). Similarly, pretreatment with risperidone resulted in a decrease in NO production to 29.28 ± 2.36 μM/L (*p* < 0.001) and haloperidol resulted in a decrease to 40.37 ± 2.36 μM/L (*p* < 0.001) compared to the LPS group.

**Fig. 6:**
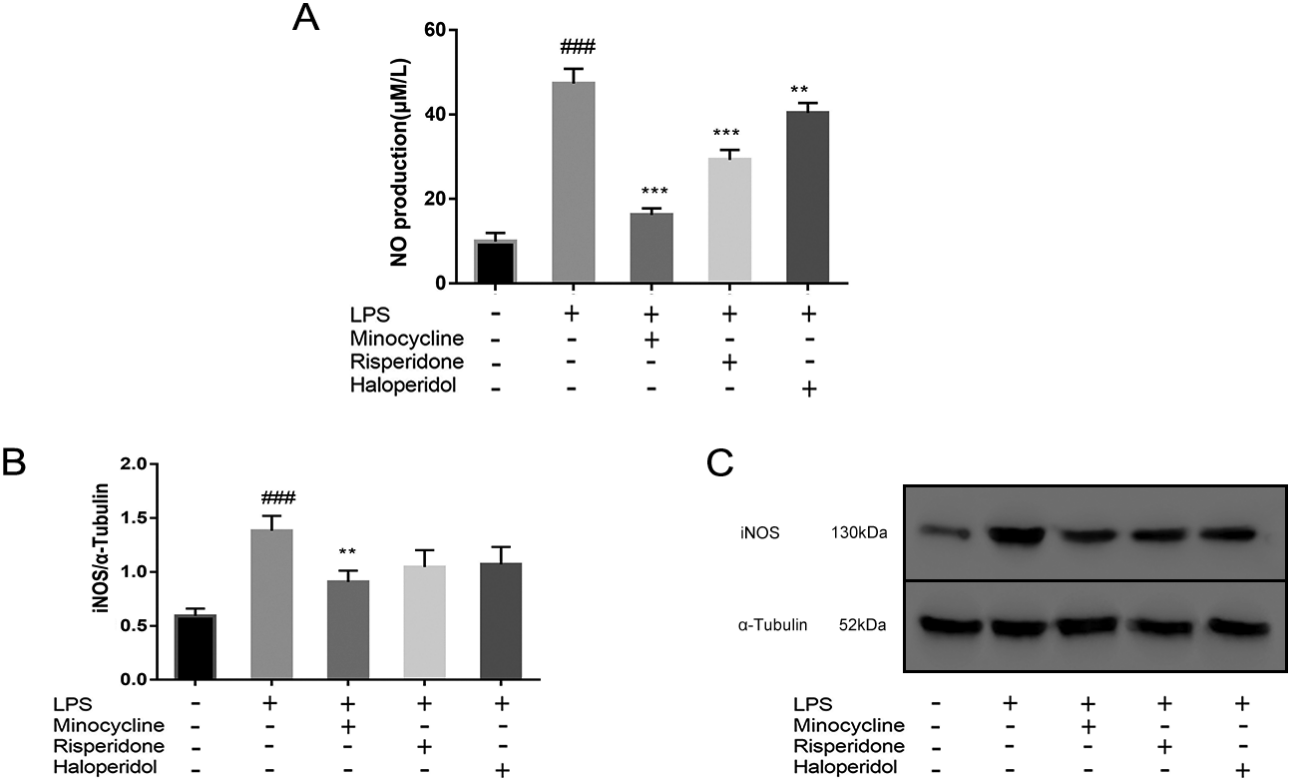
Effects of minocycline and antipsychotics on NO and iNOS expression of microglia activated by LPS. The cells were randomly divided into 5 groups. The blank control group was treated with nothing, the LPS group was treated with 1.0 ug/ml LPS for 24 h. The three drug groups were pretreated with 10 μmol/L minocycline, 10 μmol/L risperidone and 50 μmol/L haloperidol respectively for 24 h, and then 1.0 ug/ml LPS was added for 24 h. The production of NO (**A**) in conditioned medium was measured by Griess’s reagent assay, and the band density of iNOS (**B**) was used to quantify the expression level normalized with a band density of α-Tubulin. The expression of iNOS was determined by western blot analysis (**C**). ^###^*p* < 0.001 was compared with the blank control group; **p < 0.01 and ***p < 0.001 were compared with the LPS group. The original image of the full gels/blots was included in the Supplementary Fig. S1.

In order to determine whether this inhibition was related to the expression of intracellular iNOS, the expression of iNOS in each group was analyzed by western blot analysis. The results showed that only minocycline significantly inhibited the overexpression of iNOS induced by LPS (*p* < 0.01). The expression of iNOS in risperidone and haloperidol group also showed a downward trend, but there was no significant difference compared with the LPS group. (Fig. 6)

### Effects of minocycline and antipsychotics on MAPK inflammatory signaling pathway in BV-2 microglial cells

**Fig. 7:**
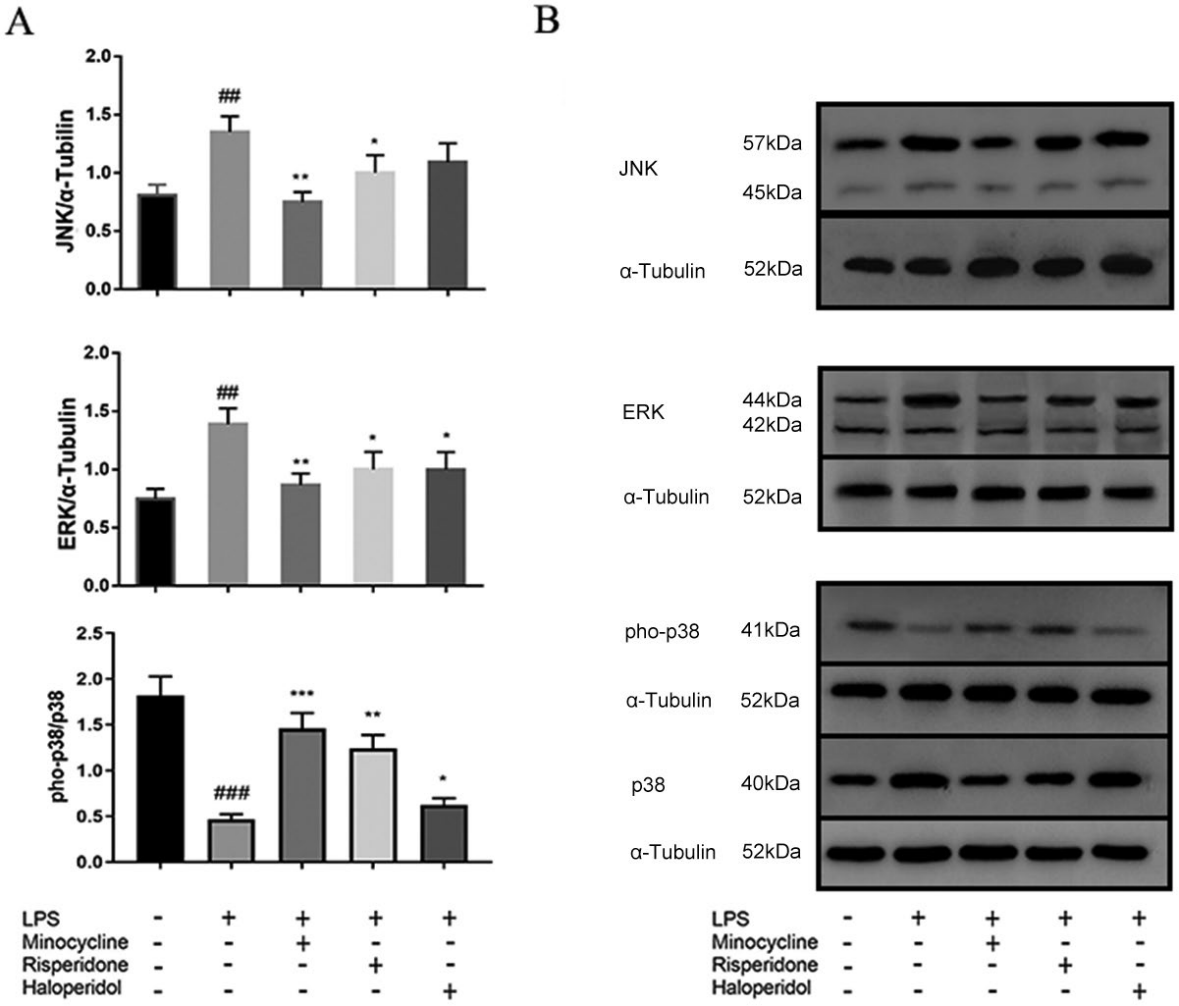
Effects of minocycline and antipsychotics on MAPKs inflammatory signaling pathway in BV-2 microglial cells. The cells were randomly divided into 5 groups. The blank control group was treated with nothing, the LPS group was treated with 1.0 ug/ml LPS for 24 h. The three drug groups were pretreated with 10 μmol/L minocycline, 10 μmol/L risperidone and 50 μmol/L haloperidol respectively for 24 h, and then 1.0 ug/ml LPS was added for 24 h. The band density was used to quantify the expression levels of JNK, ERK, and pho-p38/ p38 normalized with a band density of α-Tubulin (**A**). The expression of the proteins was determined by western blot analysis (**B**). ^##^*p* < 0.01 and ^###^*p* < 0.001 were compared with the blank control group; *p < 0.05, **p < 0.01 and ***p < 0.001 were compared with the LPS group. The original image of the full gels/blots was included in the Supplementary Fig. S2, Fig.S3, Fig.S4, and Fig.S5.

The MAPK family consists of three key members: extracellular signal-regulated kinases (ERK), c-Jun N-terminal kinases (JNK) and p38 MAPK. Administration of LPS induced a decrease in phosphorylated p38 (pho-p38) (*p* < 0.001) and increased the expression of JNK, and ERK in MAPK inflammatory signaling pathway (*p* < 0.01). Minocycline and risperidone significantly down-regulated the expression of JNK (*p* < 0.01), ERK (*p* < 0.01) and increased the phosphorylation level of p38 (*p* < 0.001 for minocycline, and *p* < 0.01 for risperidone) in BV2 cells activated by LPS, while haloperidol down-regulated the expression of ERK significantly (*p* < 0.05) and increased the phosphorylation level of p38 (*p* < 0.05) (Fig. 7).

### Effects of minocycline and antipsychotics on JAK-STAT inflammatory signaling pathway in BV-2 microglial cells

**Fig. 8:**
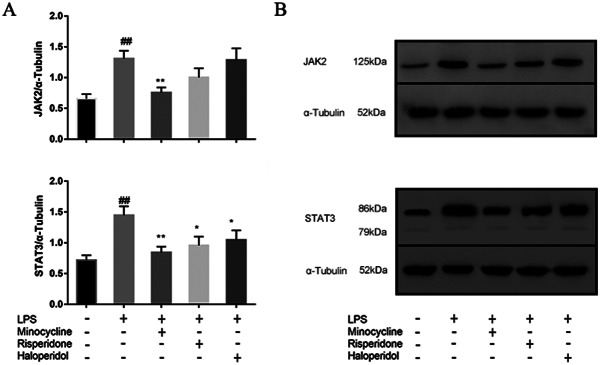
Effects of minocycline and antipsychotics on JAK2-STAT3 inflammatory signaling pathway in BV-2 microglial cells. The cells were randomly divided into 5 groups. The blank control group was treated with nothing, the LPS group was treated with 1.0 ug/ml LPS for 24 h. The three drug groups were pretreated with 10 μmol/L minocycline, 10 μmol/L risperidone and 50 μmol/L haloperidol respectively for 24 h, and then 1.0 ug/ml LPS was added for 24 h. The band density was used to quantify the expression levels of JAK2 and STAT3 normalized with a band density of α-Tubulin (**A**). The expression of the proteins was determined by western blot analysis (**B**). ^##^*p* < 0.01 was compared with the blank control group; *p < 0.05 and **p < 0.01were compared with the LPS group. The original image of the full gels/blots was included in the Supplementary Fig. S6, and Fig.S7.

To examine whether the JAK-STAT pathway was activated by LPS in microglia cells, we examined the activation of two main proteins (JAK2 and STAT3) by western blot analysis in BV2 cells. The expression of JAK2 and STAT3 were measured by western blot analysis. Data (Fig. 8) showed that administration of LPS induced an increase the expression of JAK2 (*p* < 0.01) and STAT3 (*p* < 0.01) in BV2 cells. Minocycline significantly decreased the overexpression of these two proteins activated by LPS (*p* < 0.01), while risperidone (*p* < 0.05) and haloperidol (*p* < 0.05) only down-regulated the expression of STAT3 significantly (Fig. 8).

## Discussion

Negative symptoms have long been challenging in the treatment of schizophrenia. Minocycline and atypical antipsychotics have been reported to be more effective in attenuating negative symptoms [16, 21], but the specific mechanism underlying their effects is poorly understood. In this study, we first reported that risperidone exerted stronger anti-inflammatory and neuroprotective effects than haloperidol in microglia stimulated with LPS through the MAPK and JAK-STAT signaling pathways, revealing the underlying mechanisms of minocycline and atypical antipsychotics in the treatment of negative schizophrenia symptoms.

Under LPS stimulation, microglia are activated, and their morphology changes from branched to amoeboid. Amoeboid microglia can release inflammatory mediators and proinflammatory cytokines, thus causing neuronal damage. Numerous studies [18, 22, 23] have found that antipsychotic drugs can inhibit the inflammatory response caused by microglial activation, but few studies have investigated the effects of antipsychotic drugs on the morphological changes attributed to microglial activation. In the present study, we found that minocycline and risperidone inhibited morphological changes and reduced the protein expression of OX-42 in LPS-induced activated microglia, while haloperidol did not rescue cell morphology as effectively as minocycline and risperidone. This further confirmed the inhibitory effect of antipsychotic drugs on the activation of microglia.

In vivo [10, 24] and in vitro [15, 18] studies have confirmed that minocycline and atypical antipsychotics, such as risperidone, can inhibit the production of proinflammatory cytokines in activated microglia, which is consistent with the findings of our study. The anti-inflammatory effect of typical antipsychotics in vivo has been reported. Two studies [25, 26] found that flupentixol and trifluperidol reduced the release of IL-1β, IL-2 and TNF-α by activated microglia, and chlorpromazine and loxapine [27] have also been reported to reduce the release of IL-1β and IL-2 by activated microglia. A recent study using single-cell RNA-sequencing has provided further evidence that pathways associated with microglial activation and inflammation play a role in the response to antipsychotics. The study also found that long-term exposure to atypical antipsychotic drugs, such as olanzapine, resulted in significantly more differentially expressed genes in mouse striatal microglia compared to typical antipsychotic drugs like haloperidol [28]. However, research on whether haloperidol can inhibit the release of proinflammatory cytokines by activated microglia is limited. Another recent study found that both haloperidol and risperidone reduce the pro-inflammatory action of BV2 cells [29].In the present study, we explored the anti-inflammatory effect of haloperidol in vitro and found that it had a slight anti-inflammatory effect, as it inhibited the production of IL-6 but not IL-1β and TNF-α. These data are consistent with the findings of a previous study [18] showing that risperidone significantly inhibited inflammatory responses following interferon-gamma-induced microglial activation.

NO has been reported to be related to the etiology of schizophrenia. iNOS is the key enzyme responsible for NO production and can be found in neuronal cells [30]. Clinical [31] and postmortem histochemical [32] studies have found that NO and iNOS in the brains and plasma of schizophrenia patients are expressed at higher levels than in those in the brains and plasma of healthy controls. In a neurodevelopmental model of schizophrenia, the use of minocycline was found to reduce iNOS expression in the prefrontal cortex and caudate-putamen while also preventing morphometric abnormalities in the third ventricle [33]. Both in vivo [10] and in vitro [18, 34] studies have found that atypical antipsychotics, such as risperidone and olanzapine, can inhibit the production of NO and the expression of iNOS caused by the activation of microglia. However, whether typical antipsychotics have similar effects remains unclear. In the present study, both risperidone and haloperidol inhibited the production of NO but had no significant effect on iNOS expression. The lack of effect of risperidone on the expression of iNOS may have been related to the concentration of drug administered, as the concentrations of antipsychotics used in this study were selected based on their effects on cell viability, and a concentration gradient was not used. Researchers have differing opinions on whether haloperidol can inhibit the production of NO in activated microglia [18, 34]. The results of this study suggest that haloperidol does inhibit NO production in activated microglia but that the effect of haloperidol is weaker than that of risperidone. Whether haloperidol can inhibit NO production remains to be verified by further experiments.

The MAPK signaling pathway plays an important role in regulating neuroplasticity and inflammation and is closely related to negative effects caused by the activation of microglia and the secretion of cytokines [35–37]. A study involving a model of mild neuroinflammation [10] reported that risperidone suppressed the increase in p38-MAPK expression in the prefrontal cortex. In the present study, minocycline and risperidone inhibited the LPS-induced increase of JNK, ERK and dephosphorylation of p38, which are involved in the MAPK signaling pathway, while haloperidol suppressed the activation of only ERK signaling and dephosphorylation of p38. The above results indicate that minocycline and risperidone exert anti-inflammatory effects through the same MAPK pathway, while haloperidol affects only part of the pathway.

The JAK-STAT signaling pathway, which mediates the signals that induce the early secretion of inflammatory cytokines in activated microglia and may also be closely related to the gene expression of late inflammatory mediators [38], is involved in neuroimmune regulation. Previous study [39] has found that the activation of microglia induced by ganglioside and interferon is involved in the immune inflammatory response in the central nervous system through the JAK-STAT signaling pathway. The results herein demonstrated that minocycline suppressed the activation of JAK2 and STAT3 induced by LPS, while risperidone and haloperidol suppressed the activation of only STAT3. These results suggest that the JAK-STAT signaling pathway is involved in the anti-inflammatory effects of minocycline, risperidone and haloperidol.

The limitations of our study include the use of in-vitro conditions and immortalized BV-2 microglial cells instead of primary microglial cells. While BV-2 microglial cells are commonly used for microglial studies due to their similarity to primary microglia, further research using primary cultures and in vivo settings is necessary to validate our findings.

## Conclusions

In summary, this study identified the important roles of the MAPK and JAK-STAT signaling pathways in the anti-inflammatory effect of minocycline and antipsychotics. This expands our current understanding of the mechanisms of minocycline and atypical antipsychotics in the treatment of the negative symptoms of schizophrenia.

## Electronic supplementary material

Below is the link to the electronic supplementary material.

Supplementary Material 1: The original image of the full gels/blots

## Data Availability

The datasets used and/or analysed during the current study are available from the corresponding author on reasonable request.

## List of abbreviations

CCK-8: Cell counting kit-8
ERK: Extracellular signal-regulated kinase
IL-1β: Interleukin-1beta
IL-6: Interleukin-6
iNOS: Inducible nitric oxide synthase
JAK-STAT: Janus kinase-signal transducer and activator of transcription
JAK2: Janus kinase 2
JNK: C‑Jun N‑terminal kinas
LPS: Lipopolysaccharide
MAPKs: Mitogen-activated protein kinases
MFI: Mean fluorescence intensity
NO: Nitric oxide
STAT3: Signal transducer and activator of transcription 3
TNF-α: Tumor necrosis factor-alpha

## References

[1] Fond G, Lancon C, Korchia T, Auquier P, Boyer L (2020). The role of inflammation in the treatment of Schizophrenia. Front Psychiatry.

[2] Andrea Grostøl Dietz SAG, Maiken Nedergaard (2020). Glial cells in schizophrenia: a unified hypothesis. Lancet Psychiatry.

[3] Laskaris LE, Everall MADBI, Christopoulos GChanaA, Skafidas E. VLCropley and C Pantelis. Microglial activation and progressive brain changes in schizophrenia. Br J Pharmacol 2016; (173):666–80. 10.1111/bph.13364 PMC4742288 26455353

[4] De Picker LJ, Morrens M, Chance SA, Boche D (2017). Microglia and Brain plasticity in Acute psychosis and Schizophrenia Illness Course: a Meta-review. Front Psychiatry.

[5] Rodrigues-Neves AC, Ambrósio AF, Gomes CA (2022). Microglia sequelae: brain signature of innate immunity in schizophrenia. Transl Psychiatry.

[6] Al-Samhari MM, Al-Rasheed NM, Al-Rejaie S, Al-Rasheed NM, Hasan IH, Mahmoud AM (2016). Possible involvement of the JAK/STAT signaling pathway in N-acetylcysteine-mediated antidepressant-like effects. Exp Biol Med (Maywood).

[7] Nicolas CS, Peineau S, Amici M, Csaba Z, Fafouri A, Javalet C (2012). The Jak/STAT pathway is involved in synaptic plasticity. Neuron.

[8] Shariq AS, Brietzke E, Rosenblat JD, Pan Z, Rong C, Ragguett RM (2018). Therapeutic potential of JAK/STAT pathway modulation in mood disorders. Rev Neurosci.

[9] Zhang J, He H, Qiao Y, Zhou T, He H, Yi S (2020). Priming of microglia with IFN-gamma impairs adult hippocampal neurogenesis and leads to depression-like behaviors and cognitive defects. Glia.

[10] MacDowell KS, Garcia-Bueno B, Madrigal JL, Parellada M, Arango C, Mico JA (2013). Risperidone normalizes increased inflammatory parameters and restores anti-inflammatory pathways in a model of neuroinflammation. Int J Neuropsychopharmacol.

[11] Tiina Tikka BLF, Gundars, Goldsteins (2001). Riitta Keina¨nen, and Jari Koistinaho. Minocycline, a Tetracycline Derivative, is neuroprotective against excitotoxicity by inhibiting activation and proliferation of Microglia. J Neurosci.

[12] Naura AS, Kim H, Ju J, Rodriguez PC, Jordan J, Catling AD (2013). Minocycline blocks asthma-associated inflammation in part by interfering with the T cell receptor-nuclear factor kappaB-GATA-3-IL-4 axis without a prominent effect on poly(ADP-ribose) polymerase. J Biol Chem.

[13] Chaudhry IB, Hallak J, Husain N, Minhas F, Stirling J, Richardson P (2012). Minocycline benefits negative symptoms in early schizophrenia: a randomised double-blind placebo-controlled clinical trial in patients on standard treatment. J Psychopharmacol.

[14] Shin H, Song JH (2014). Antipsychotics, chlorpromazine and haloperidol inhibit voltage-gated proton currents in BV2 microglial cells. Eur J Pharmacol.

[15] Bian Q, Kato T, Monji A, Hashioka S, Mizoguchi Y, Horikawa H (2008). The effect of atypical antipsychotics, perospirone, ziprasidone and quetiapine on microglial activation induced by interferon-gamma. Prog Neuropsychopharmacol Biol Psychiatry.

[16] Zhu F, Zheng Y, Ding YQ, Liu Y, Zhang X, Wu R (2014). Minocycline and risperidone prevent microglia activation and rescue behavioral deficits induced by neonatal intrahippocampal injection of lipopolysaccharide in rats. PLoS ONE.

[17] Zhu F, Zheng Y, Liu Y, Zhang X, Zhao J (2014). Minocycline alleviates behavioral deficits and inhibits microglial activation in the offspring of pregnant mice after administration of polyriboinosinic-polyribocytidilic acid. Psychiatry Res.

[18] Kato T, Monji A, Hashioka S, Kanba S (2007). Risperidone significantly inhibits interferon-gamma-induced microglial activation in vitro. Schizophr Res.

[19] Kato TA, Monji A, Mizoguchi Y, Hashioka S, Horikawa H, Seki Y (2011). Anti-inflammatory properties of antipsychotics via microglia modulations: are antipsychotics a ‘fire extinguisher’ in the brain of schizophrenia?. Mini Rev Med Chem.

[20] Caruso G, Grasso M, Fidilio A, Tascedda F, Drago F, Caraci F. Antioxidant Properties of Second-Generation Antipsychotics: Focus on Microglia. Pharmaceuticals (Basel). 2020; 13(12). 10.3390/ph13120457 PMC7764768 33322693

[21] Xiang YQ, Zheng W, Wang SB, Yang XH, Cai DB, Ng CH (2017). Adjunctive minocycline for schizophrenia: a meta-analysis of randomized controlled trials. Eur Neuropsychopharmacol.

[22] Zhu L, Liu X, Nemeth DP, DiSabato DJ, Witcher KG, McKim DB (2019). Interleukin-1 causes CNS inflammatory cytokine expression via endothelia-microglia bi-cellular signaling. Brain Behav Immun.

[23] Kato TA, Monji A, Yasukawa K, Mizoguchi Y, Horikawa H, Seki Y (2011). Aripiprazole inhibits superoxide generation from phorbol-myristate-acetate (PMA)-stimulated microglia in vitro: implication for antioxidative psychotropic actions via microglia. Schizophr Res.

[24] Sugino H, Futamura T, Mitsumoto Y, Maeda K, Marunaka Y (2009). Atypical antipsychotics suppress production of proinflammatory cytokines and up-regulate interleukin-10 in lipopolysaccharide-treated mice. Prog Neuropsychopharmacol Biol Psychiatry.

[25] Kowalski J, Labuzek K, Herman ZS (2003). Flupentixol and trifluperidol reduce secretion of tumor necrosis factor-alpha and nitric oxide by rat microglial cells. Neurochem Int.

[26] Kowalski J, Labuzek K, Herman ZS (2004). Flupentixol and trifluperidol reduce interleukin-1 beta and interleukin-2 release by rat mixed glial and microglial cell cultures. Pol J Pharmacol.

[27] Labuzek K, Kowalski J, Gabryel B, Herman ZS (2005). Chlorpromazine and loxapine reduce interleukin-1beta and interleukin-2 release by rat mixed glial and microglial cell cultures. Eur Neuropsychopharmacol.

[28] Abrantes A, Giusti-Rodriguez P, Ancalade N, Sekle S, Basiri ML, Stuber GD (2022). Gene expression changes following chronic antipsychotic exposure in single cells from mouse striatum. Mol Psychiatry.

[29] Racki V, Marcelic M, Stimac I, Petric D, Kucic N. Effects of Haloperidol, Risperidone, and Aripiprazole on the Immunometabolic Properties of BV-2 microglial cells. Int J Mol Sci. 2021; 22(9). 10.3390/ijms22094399 PMC8122792 33922377

[30] Mayer B, Schmidt K, Humbert P (1989). Biosynthesis of endotheliumderived relaxing factor: a cytosolic enzyme in porcine aortic endothelial cells Ca2+-dependently converts L-arginine into an activator of soluble guanylyl cyclase. Biochem Biophys Res Commun.

[31] Yanik MVH, Kocyigit A, Tutkun H, Zoroglu SS, Herken H (2003). Is the arginine-nitric oxide pathway involved in the pathogenesis of schizophrenia?. Neuropsychobiology.

[32] Akbarian S, Vinuela A, Kim JJ, Potkin SG, Bunney WE (1993). Distorted distribution of nicotinamide-adenine dinucleotide phosphate-diaphorase neurons in temporal lobe of schizophrenics implies anomalous cortical development. Arch Gen Psychiatry.

[33] Romero-Miguel D, Casquero-Veiga M, MacDowell KS, Torres-Sanchez S, Garcia-Partida JA, Lamanna-Rama N (2021). A characterization of the Effects of Minocycline Treatment during Adolescence on Structural, metabolic, and oxidative stress parameters in a maternal Immune Stimulation Model of Neurodevelopmental Brain Disorders. Int J Neuropsychopharmacol.

[34] Hou Y, Wu CF, Yang JY, He X, Bi XL, Yu L (2006). Effects of clozapine, olanzapine and haloperidol on nitric oxide production by lipopolysaccharide-activated N9 cells. Prog Neuropsychopharmacol Biol Psychiatry.

[35] Bodles AM, Barger SW (2005). Secreted beta-amyloid precursor protein activates microglia via JNK and p38-MAPK. Neurobiol Aging.

[36] Hua Fan P-FW, Zhang L, Wen Z-LH, Guan WXin-Lei, Luo H, Ni M, Yang J-W, Li M-X, Chen J-G. Fang Methionine Sulfoxide reductase A negatively controls Microglia-mediated Neuroinflammation via inhibiting ROS/MAPKs/NF-κB signaling pathways through a Catalytic antioxidant function. Volume 22. ANTIOXIDANTS & REDOX SIGNALING; 2015. pp. 832–47. 10. 10.1089/ars.2014.6022 PMC4367238 25602783

[37] Wang X, Hu D, Zhang L, Lian G, Zhao S, Wang C (2014). Gomisin A inhibits lipopolysaccharide-induced inflammatory responses in N9 microglia via blocking the NF-kappaB/MAPKs pathway. Food Chem Toxicol.

[38] Huang C, Ma R, Sun S, Wei G, Fang Y, Liu R (2008). JAK2-STAT3 signaling pathway mediates thrombin-induced proinflammatory actions of microglia in vitro. J Neuroimmunol.

[39] Kim OS, Park EJ, Joe EH, Jou I (2002). JAK-STAT signaling mediates gangliosides-induced inflammatory responses in brain microglial cells. J Biol Chem.

