# Supplementary material for: Minocycline and antipsychotics inhibit inflammatory responses in BV-2 microglia activated by LPS via regulating the MAPKs/ JAK-STAT signaling pathway
Source: BMC Psychiatry. 2023 Jul 18;23:514. doi: 10.1186/s12888-023-05014-1 (PMC10354898; doi:10.1186/s12888-023-05014-1)

Supplemental information

Supplemental figures

Fig.S1 Full-length gels and western blots for iNOS and α-Tubulin in the different treatment groups. This supplemental figure corresponds to the western blots in Fig.6C. Abbreviation: LPS lipopolysaccharide; Mino Minocycline; Ris Risperidone; Halo Haloperidol.

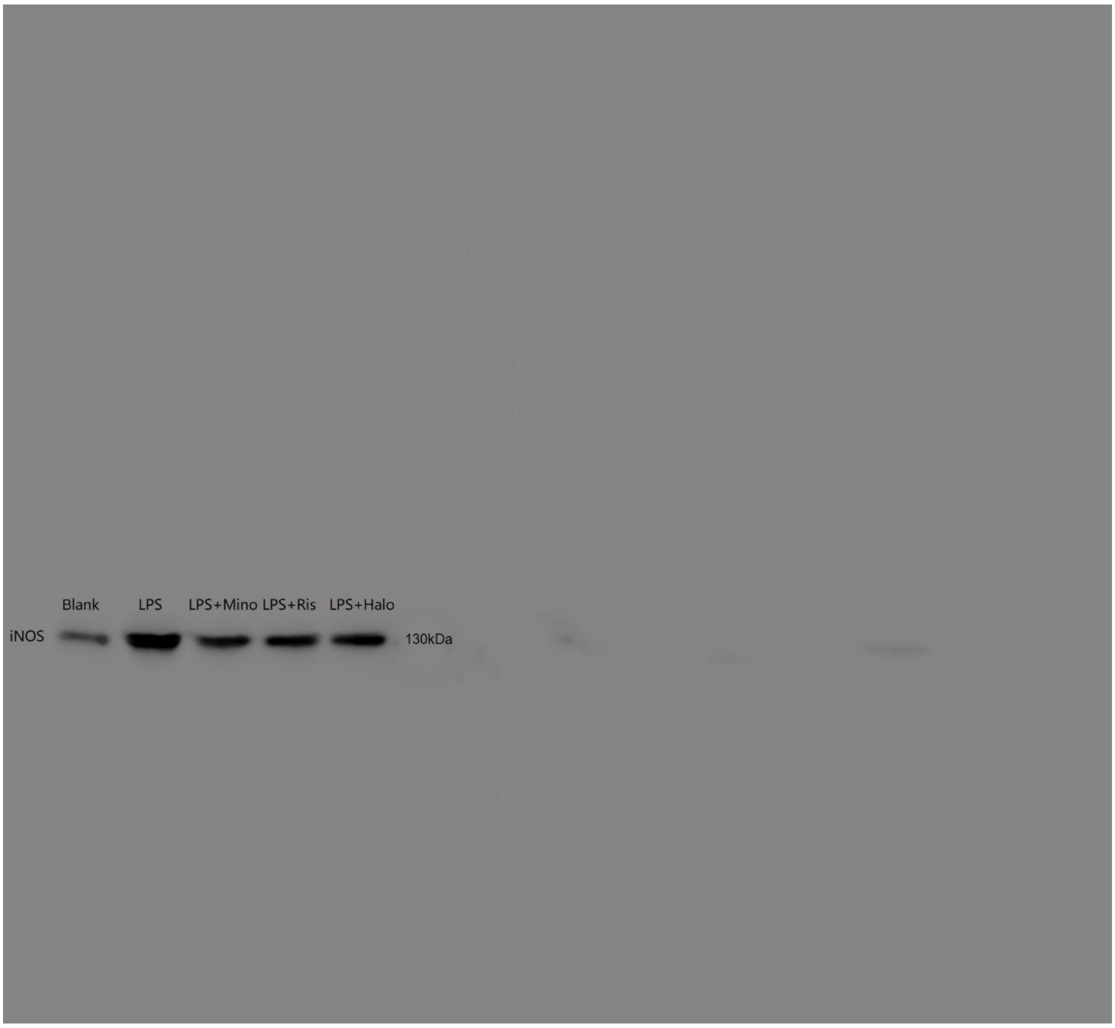

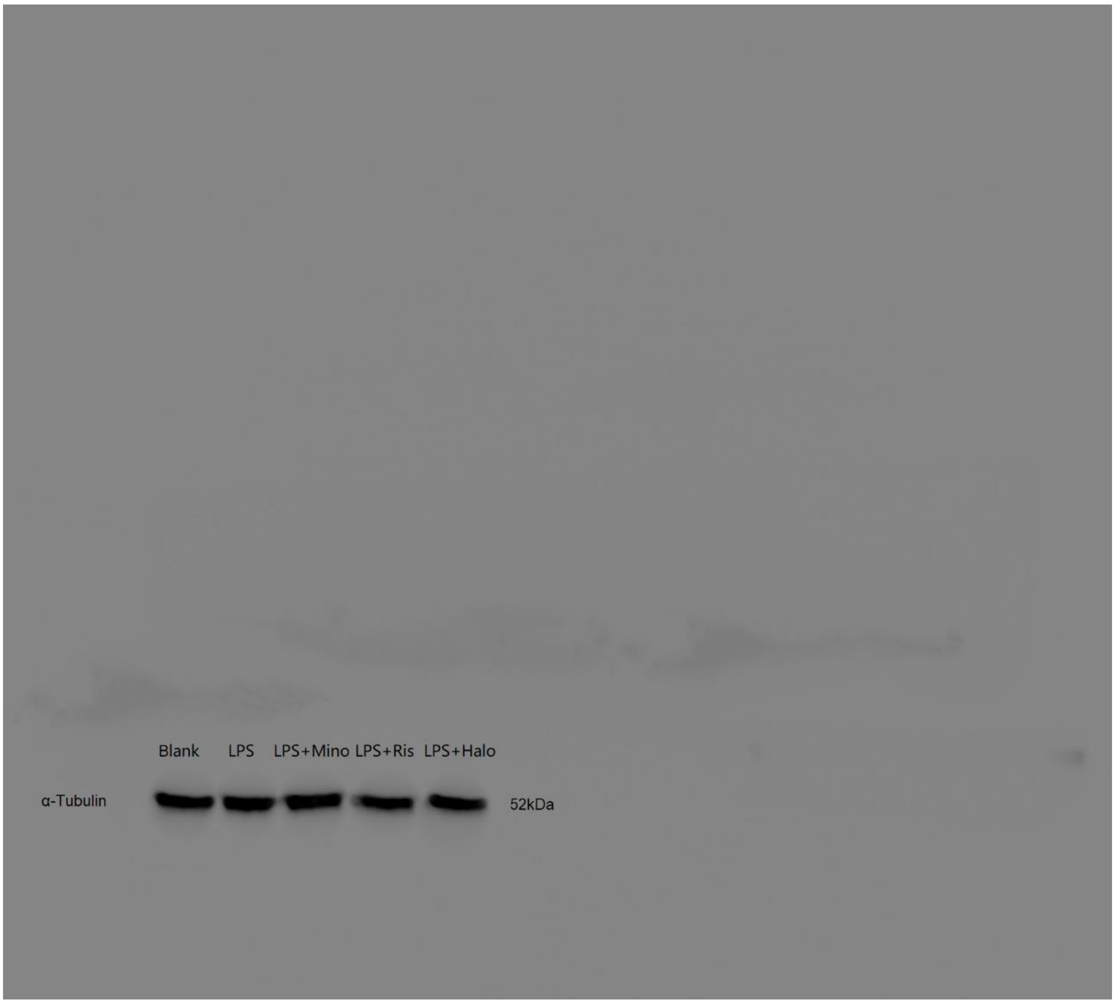

8
9

Fig.S2 Full-length gels and western blots for JNK and α-Tubulin in the different treatment groups. This supplemental figure corresponds to the western blots in Fig. 7B. Abbreviation: LPS lipopolysaccharide; Mino Minocycline; Ris Risperidone; Halo Haloperidol.

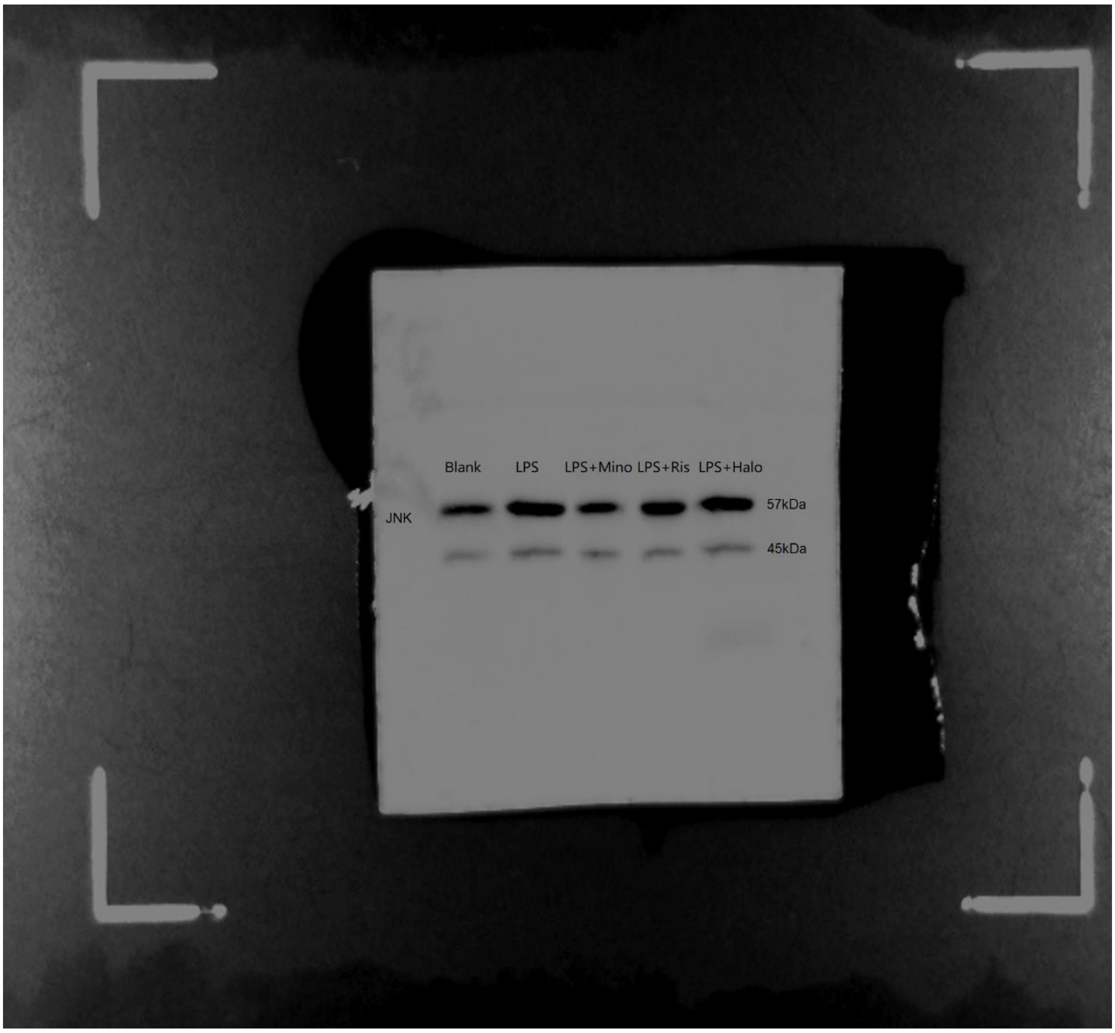

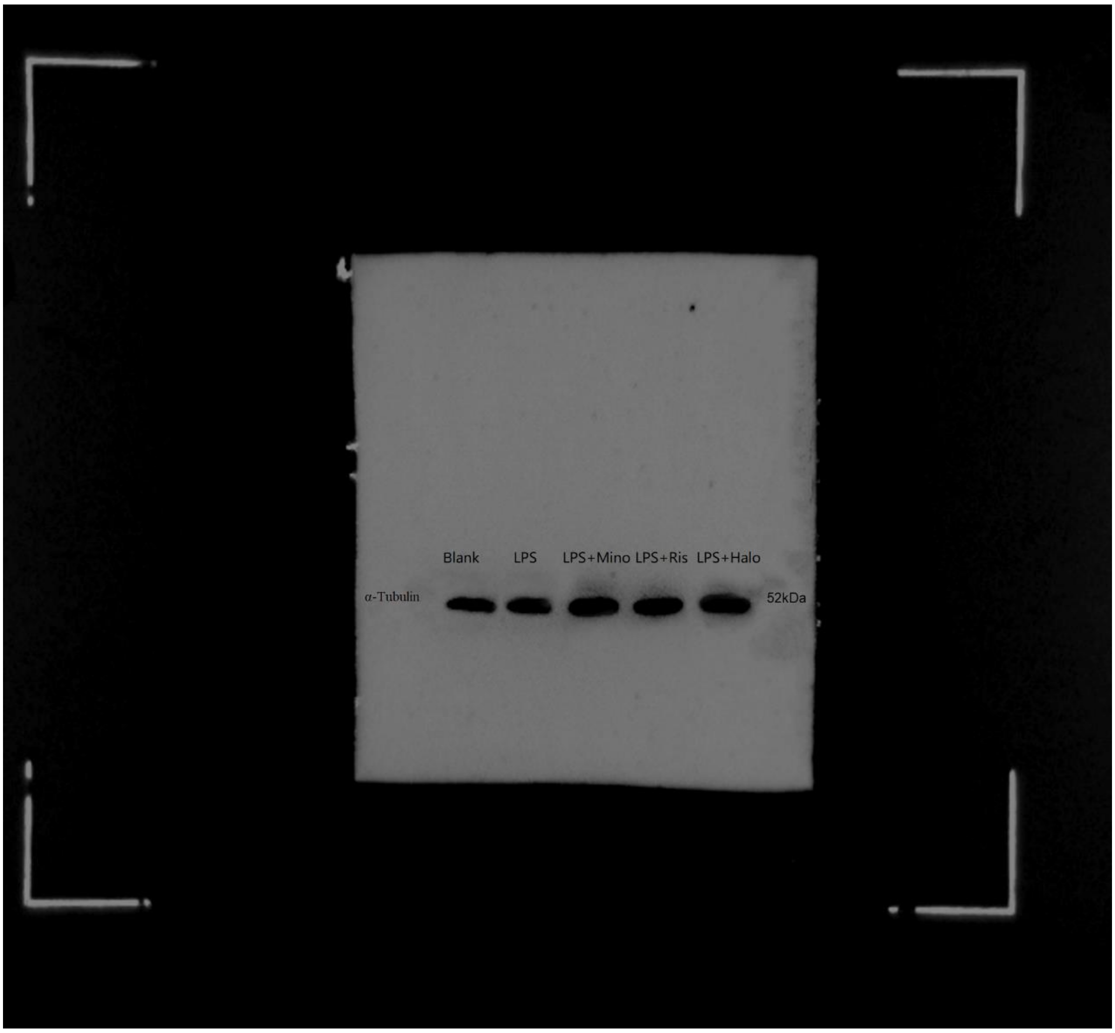
Blank
LPS
LPS+Mino
LPS+Ris
LPS+Halo
α-Tubulin
52kDa

Fig. S3 Full-length gels and western blots for ERK and α-Tubulin in the different treatment groups. This supplemental figure corresponds to the western blots in Fig. 7B. Abbreviation: LPS lipopolysaccharide; Mino Minocycline; Ris Risperidone; Halo Haloperidol.

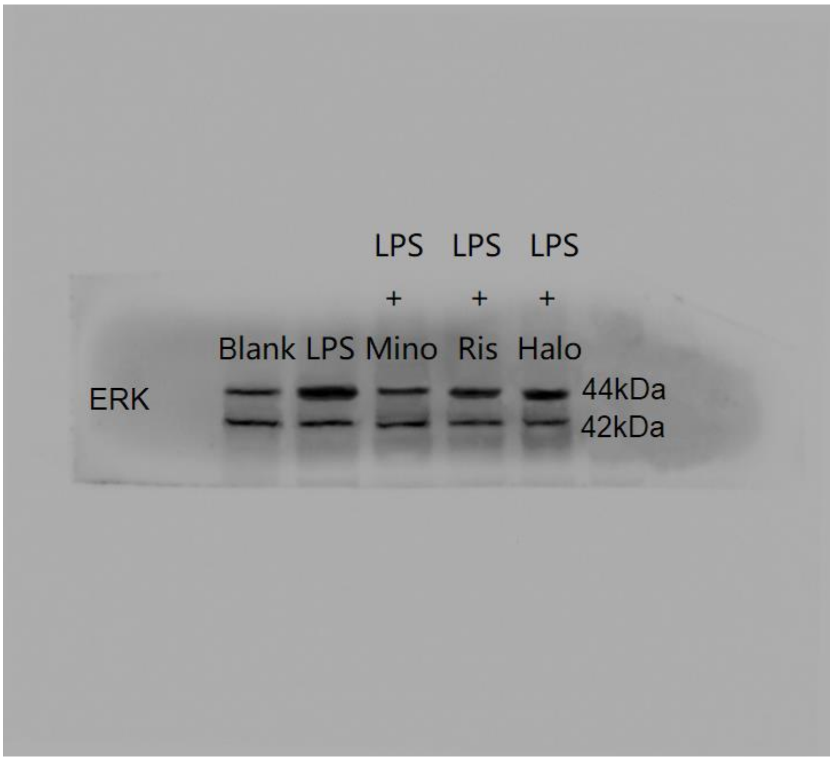

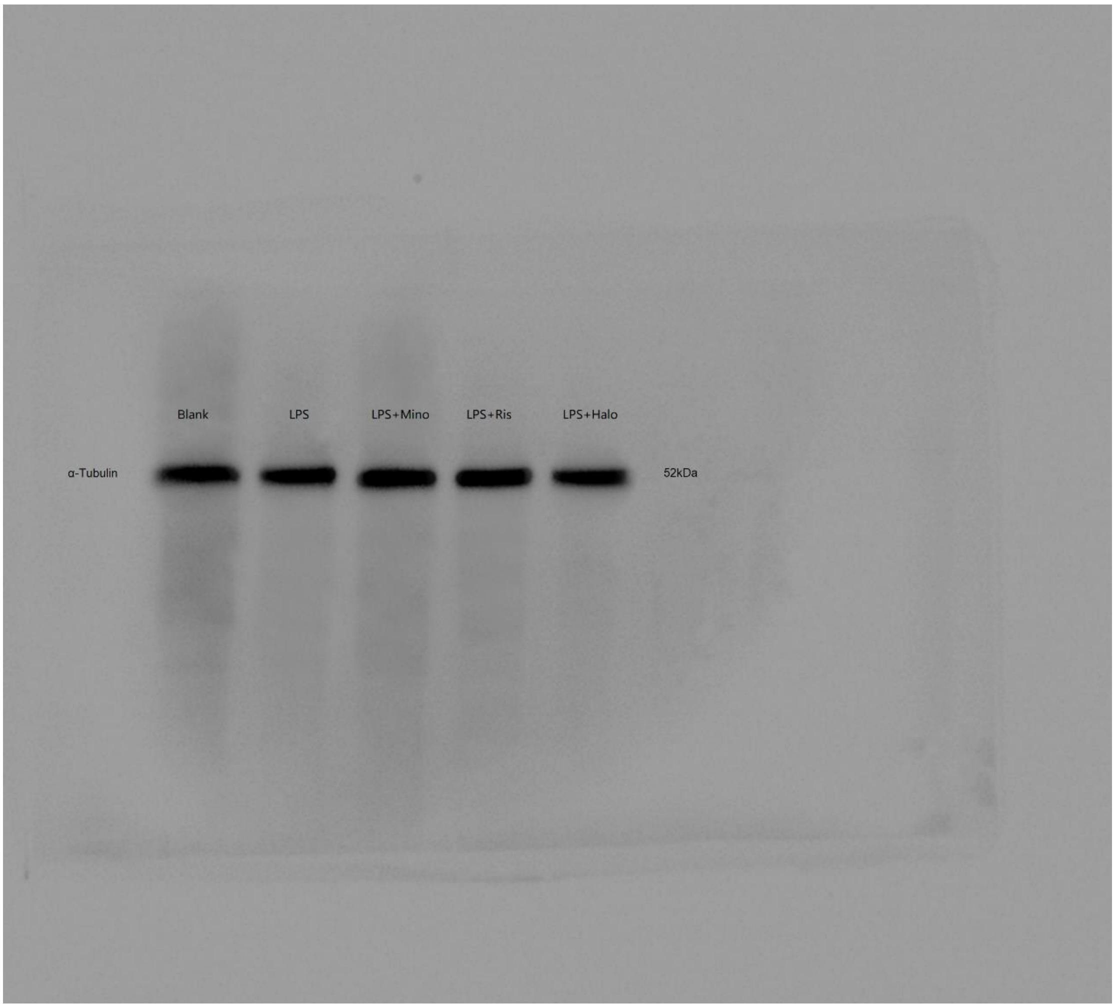
Blank
LPS
LPS+Mino
LPS+Ris
LPS+Halo
α-Tubulin
52kDa

Fig.S4 Full-length gels and western blots for pho-p38 and α-Tubulin in the different treatment groups. This supplemental figure corresponds to the western blots in Fig. 7B. Abbreviation: LPS lipopolysaccharide; Mino Minocycline; Ris Risperidone; Halo Haloperidol.

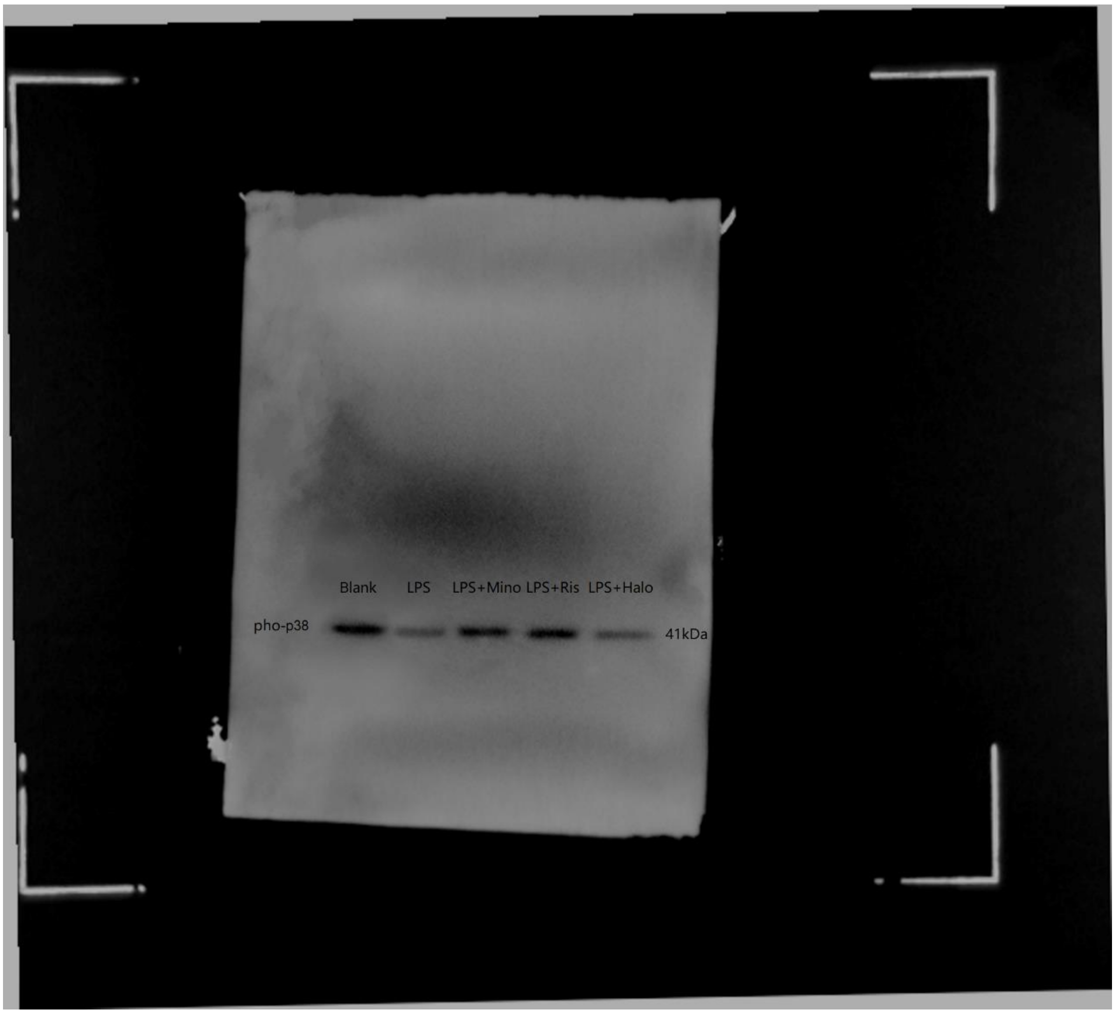

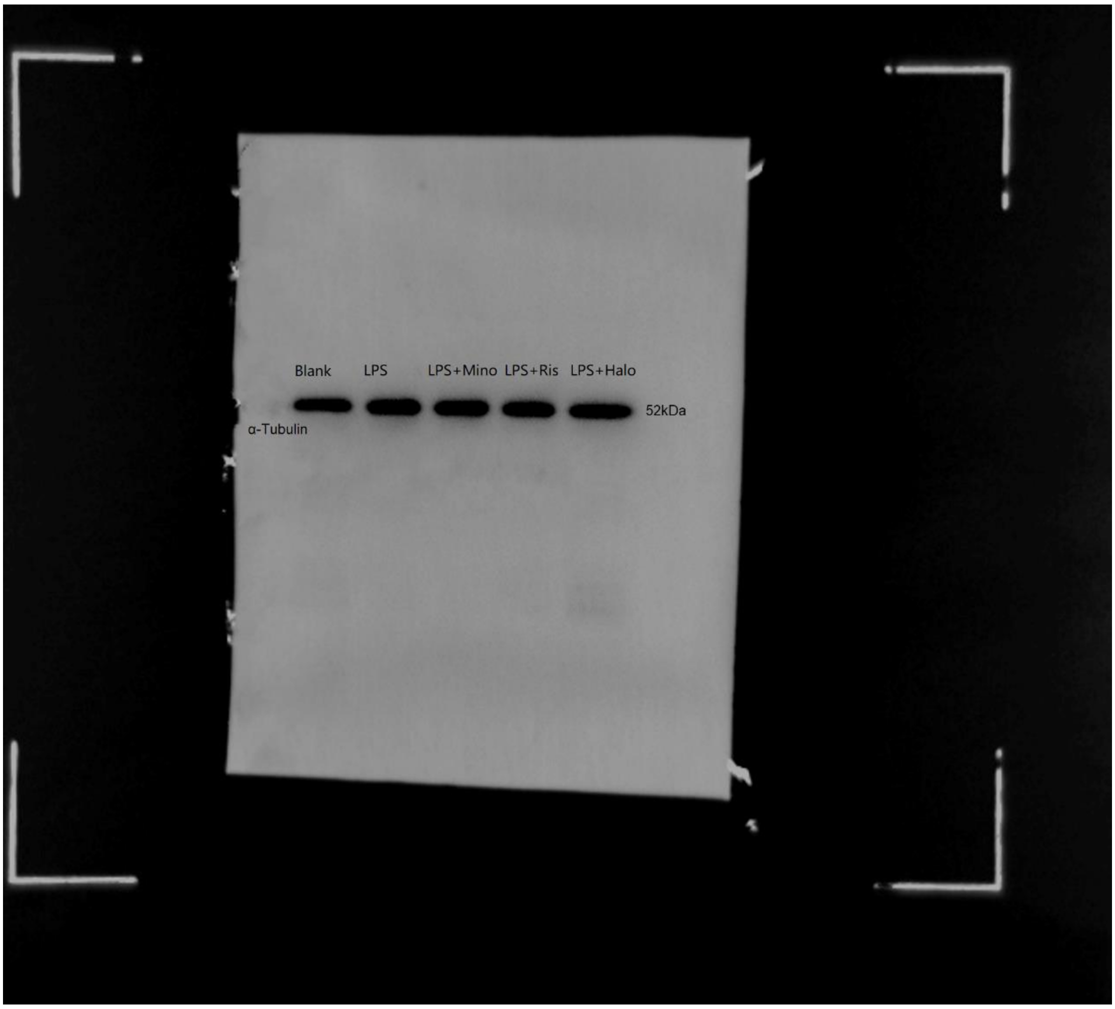
Blank
LPS
LPS+Mino
LPS+Ris
LPS+Halo
52kDa
α-Tubulin

Fig.S5 Full-length gels and western blots for p38 and α-Tubulin in the different treatment groups. This supplemental figure corresponds to the western blots in Fig. 7B. Abbreviation: LPS lipopolysaccharide; Mino Minocycline; Ris Risperidone; Halo Haloperidol.

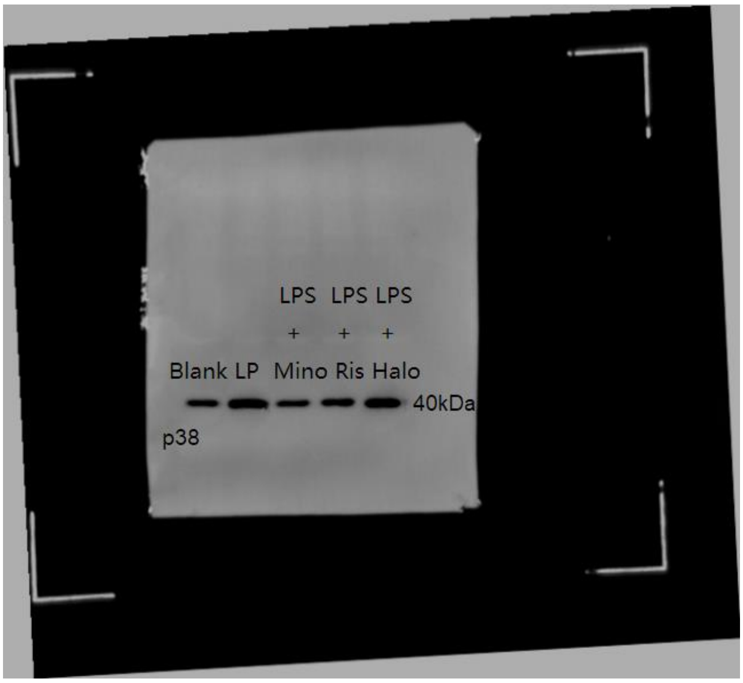

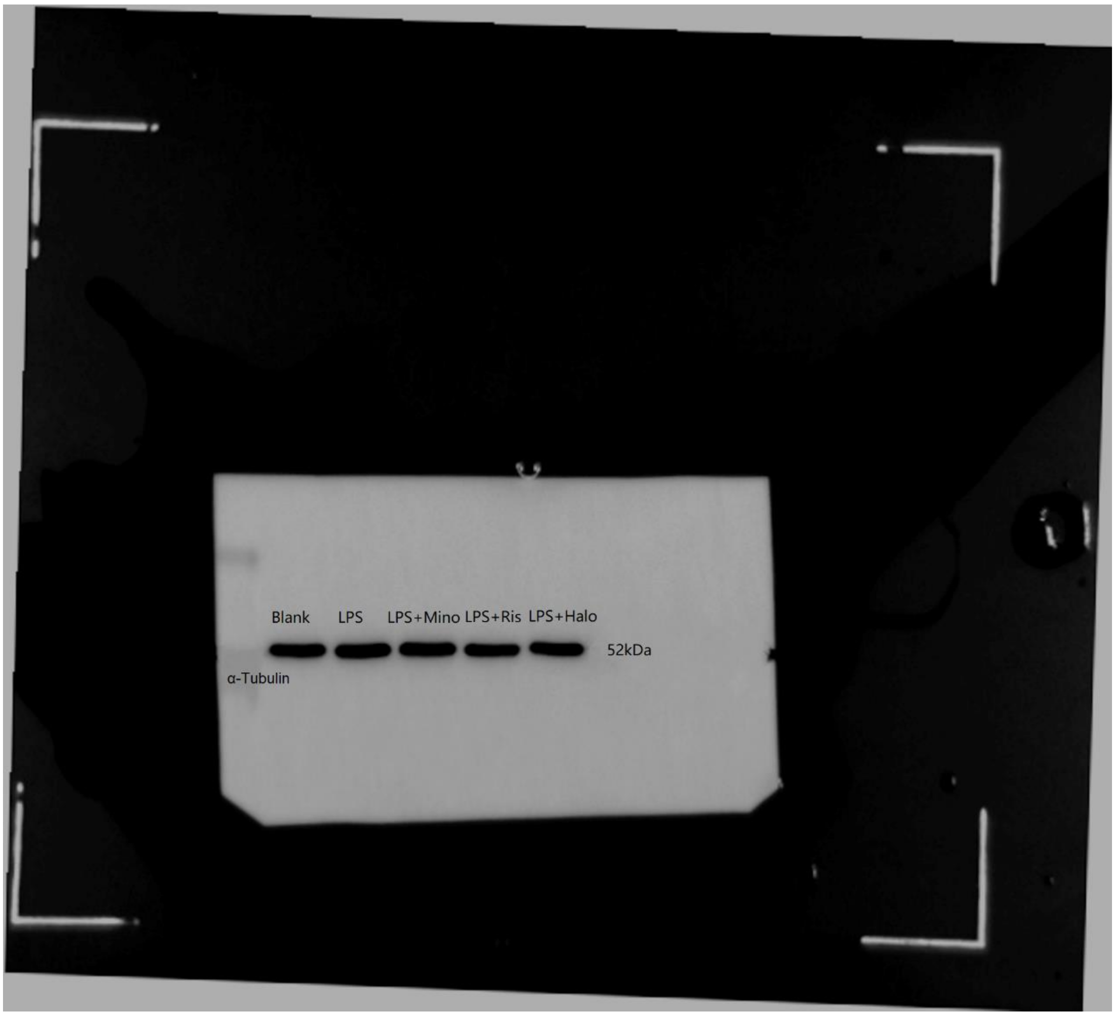
Blank
LPS
LPS+Mino
LPS+Ris
LPS+Halo
52kDa
α-Tubulin

Fig.S6 Full-length gels and western blots for JAK2 and α-Tubulin in the different treatment groups. This supplemental figure corresponds to the western blots in Fig. 8B. Abbreviation: LPS lipopolysaccharide; Mino Minocycline; Ris Risperidone; Halo Haloperidol.

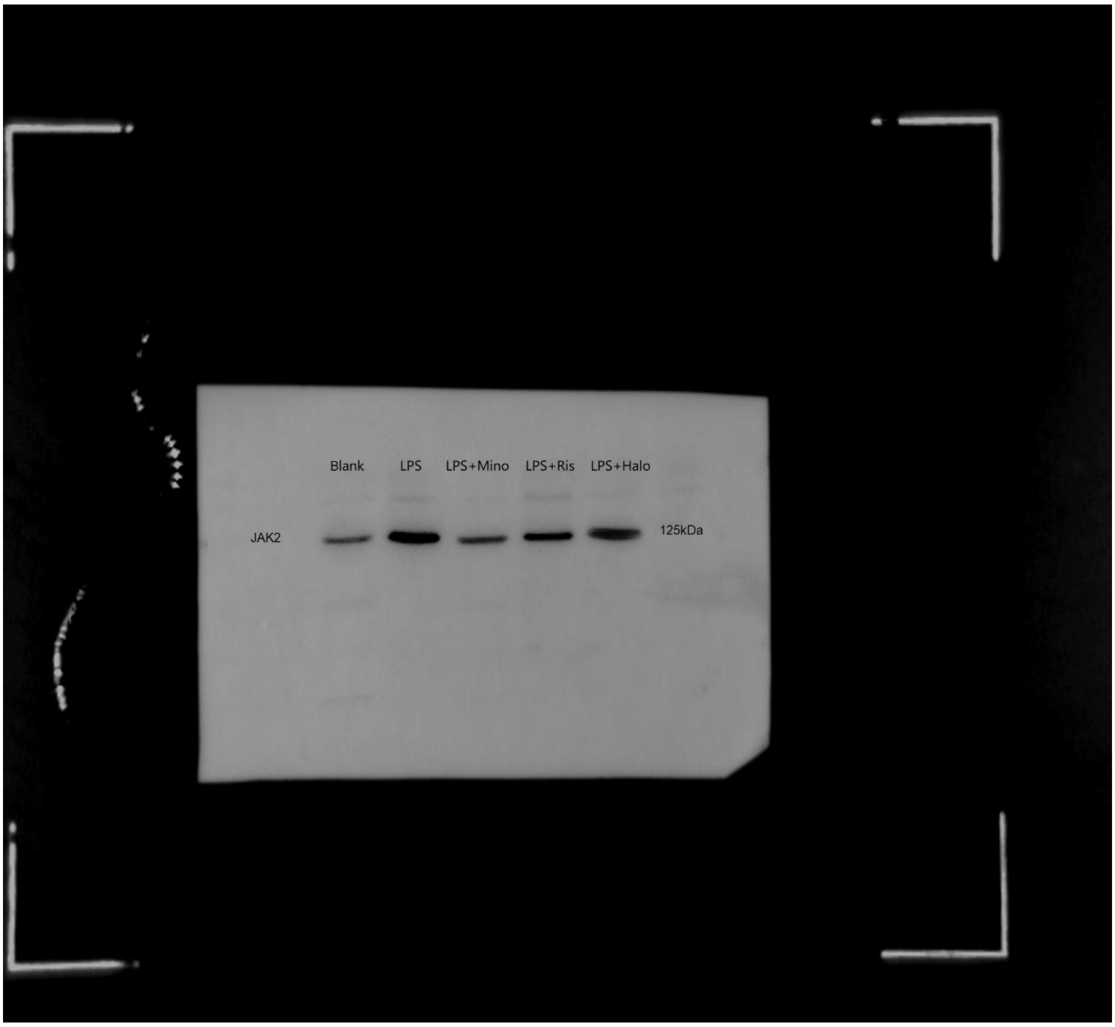

43 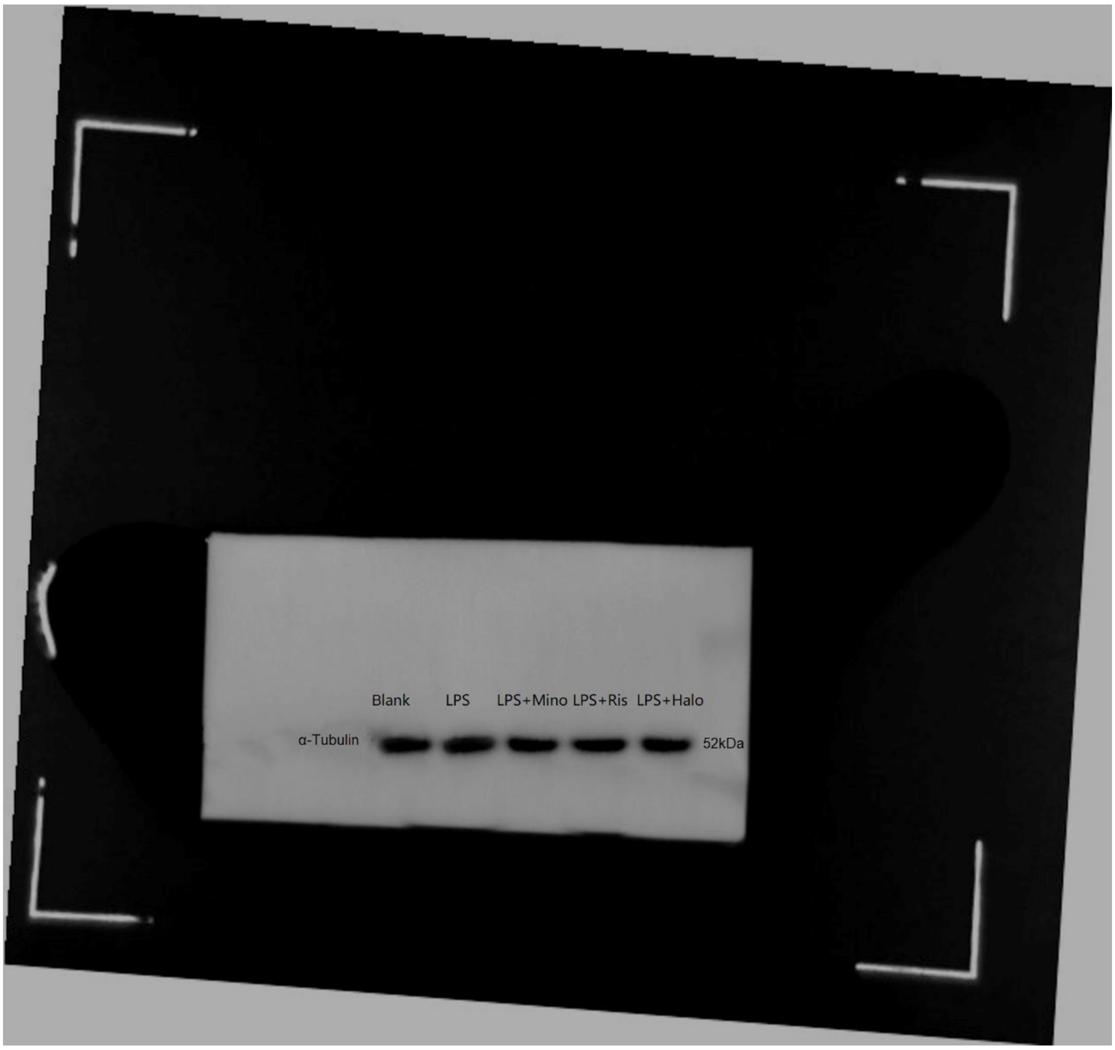

44

Fig.S7 Full-length gels and western blots for STAT3 and α-Tubulin in the different treatment groups. This supplemental figure corresponds to the western blots in Fig. 8B. Abbreviation: LPS lipopolysaccharide; Mino Minocycline; Ris Risperidone; Halo Haloperidol.

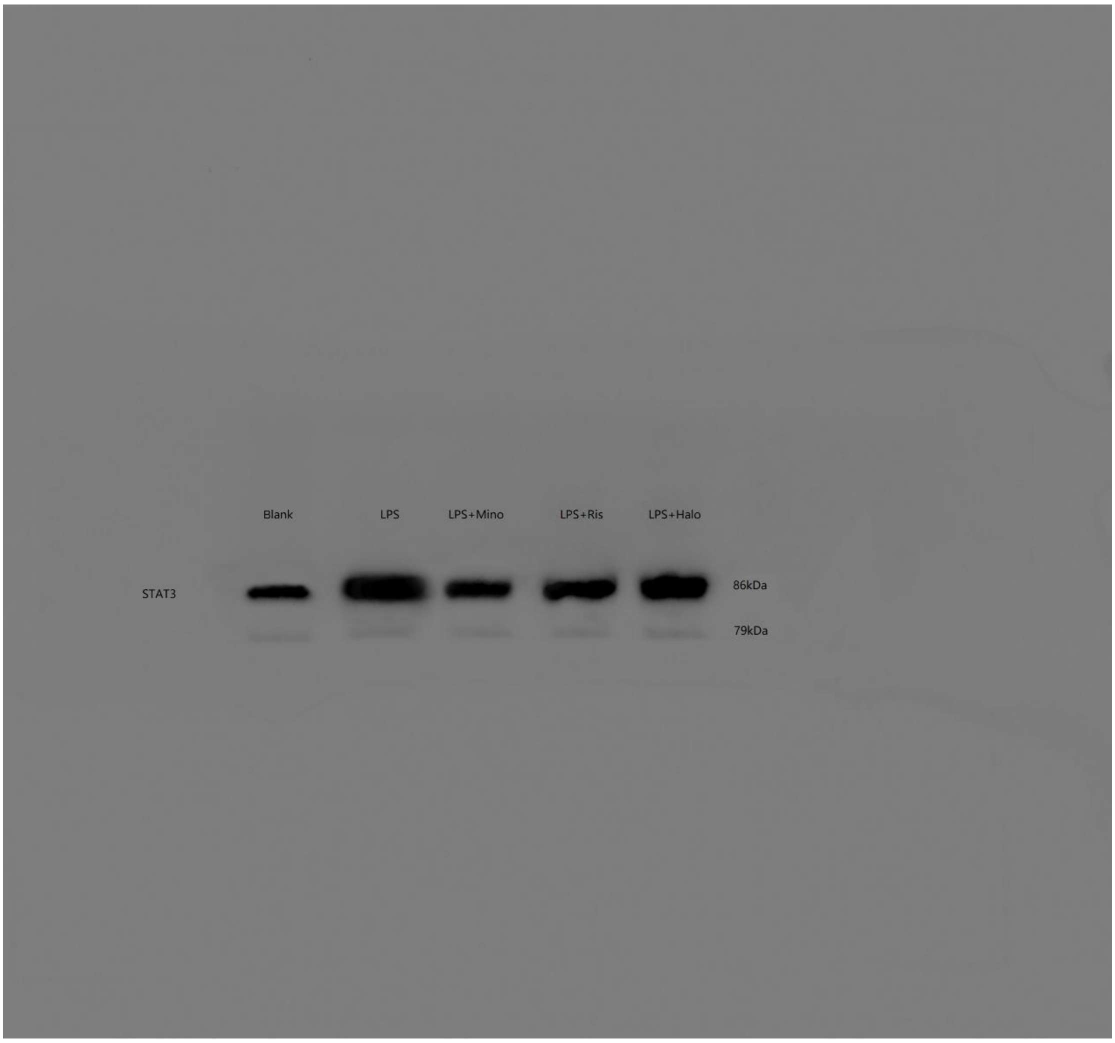

50

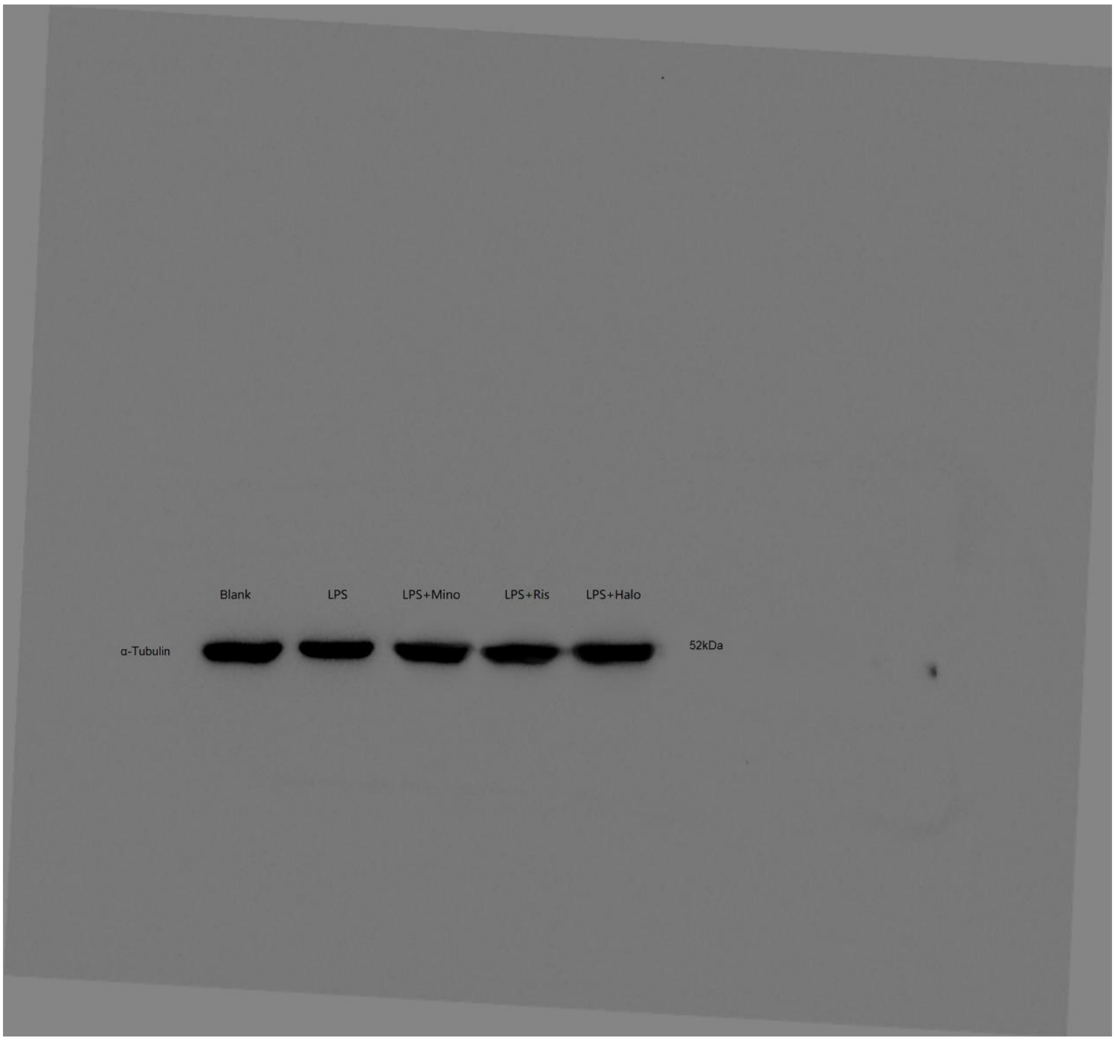

Supplement: Supplementary file 1 — Supplementary Material 1: The original image of the full gels/blots [file 12888_2023_5014_MOESM1_ESM.pdf]
